# Axial Spondyloarthritis: Mimics and Pitfalls of Imaging Assessment

**DOI:** 10.3389/fmed.2021.658538

**Published:** 2021-04-22

**Authors:** António Proença Caetano, Vasco V. Mascarenhas, Pedro M. Machado

**Affiliations:** ^1^Radiology Department, Hospital de Curry Cabral, Centro Hospitalar Universitário Lisboa Central, Lisbon, Portugal; ^2^Musculoskeletal Imaging Unit, Grupo Luz Saúde, Radiology Department, Imaging Center, Hospital da Luz, Lisbon, Portugal; ^3^EpiDoC Unit, Chronic Diseases Research Centre, NOVA Medical School, Lisbon, Portugal; ^4^Centre for Rheumatology & Department of Neuromuscular Diseases, University College London, London, United Kingdom; ^5^National Institute for Health Research (NIHR) Biomedical Research Centre, University College London Hospitals National Health Service Foundation Trust, London, United Kingdom; ^6^Department of Rheumatology, London North West University Healthcare National Health Service Trust, London, United Kingdom

**Keywords:** axial spondyloarthritis, magnetic resonance imaging, radiography, computed tomography, differential diagnosis, pitfall, normal variant, mimic

## Abstract

Axial spondyloarthritis (axSpA) is a chronic inflammatory disorder that predominantly involves the axial skeleton. Imaging findings of axSpA can be divided into active changes, which include bone marrow edema, synovitis, enthesitis, capsulitis, and intra-articular effusion, and structural changes, which include erosions, sclerosis, bone fatty infiltration, fat deposition in an erosion cavity, and bone bridging or ankylosis. The ability to distinguish between imaging lesions suggestive of axSpA and artifacts or lesions suggestive of other disorders is critical for the accurate diagnosis of axSpA. Diagnosis may be challenging, particularly in early-stage disease and magnetic resonance imaging (MRI) plays a key role in the detection of subtle or inflammatory changes. MRI also allows the detection of structural changes in the subchondral bone marrow that are not visible on conventional radiography and is of prognostic and monitoring value. However, bone structural changes are more accurately depicted using computed tomography. Conventional radiography, on the other hand, has limitations, but it is easily accessible and may provide insight on gross changes as well as rule out other pathological features of the axial skeleton. This review outlines the imaging evaluation of axSpA with a focus on imaging mimics and potential pitfalls when assessing the axial skeleton.

## Introduction

Axial spondyloarthritis (axSpA) is an umbrella term encompassing a group of chronic immune-mediated inflammatory diseases of the axial skeleton. This group includes patients with radiographic axSpA, with established sacroiliitis on radiographs, and a further subgroup called non-radiographic axSpA, who typically have evidence of sacroiliitis on magnetic resonance imaging (MRI) in the absence of definite radiographic changes.

Historically, the diagnosis of axSpA has often been delayed since radiographic abnormalities may take years to develop. Computed tomography (CT) allows for detection of smaller structural lesions in patients with chronic sacroiliitis that would otherwise be invisible on conventional radiography, thus aiding in the diagnostic work up of axSpA. In recent years, the introduction of MRI into clinical practice has facilitated earlier diagnosis of axSpA, and therefore earlier initiation of appropriate treatment. The Assessment of Spondyloarthritis International Society (ASAS) MRI working group has recently generated a consensus update on standardized definitions for MRI lesions in the sacroiliac joint (SIJ) of patients with axSpA (1). Multi-reader validation performed by the working group demonstrated substantial reliability for the most frequently detected lesions and comparable reliability between active and structural lesions. A similar exercise has been conducted for spine lesions and recently published in abstract format (2). The new consensus definitions for MRI lesions in the spine will replace a previous consensus manuscript by the same group (3).

Importantly, the full range and combination of active and structural lesions of the SIJ and spine should be taken into account when deciding if the MRI scan is suggestive of axSpA or not (i.e., contextual interpretation of active and structural lesions is key to enhancing diagnostic utility of MRI in patients with suspected axSpA), as imaging cannot be viewed in isolation and needs to be interpreted in the light of clinical presentation and results of laboratory investigations (4, 5).

**Figure 1:**
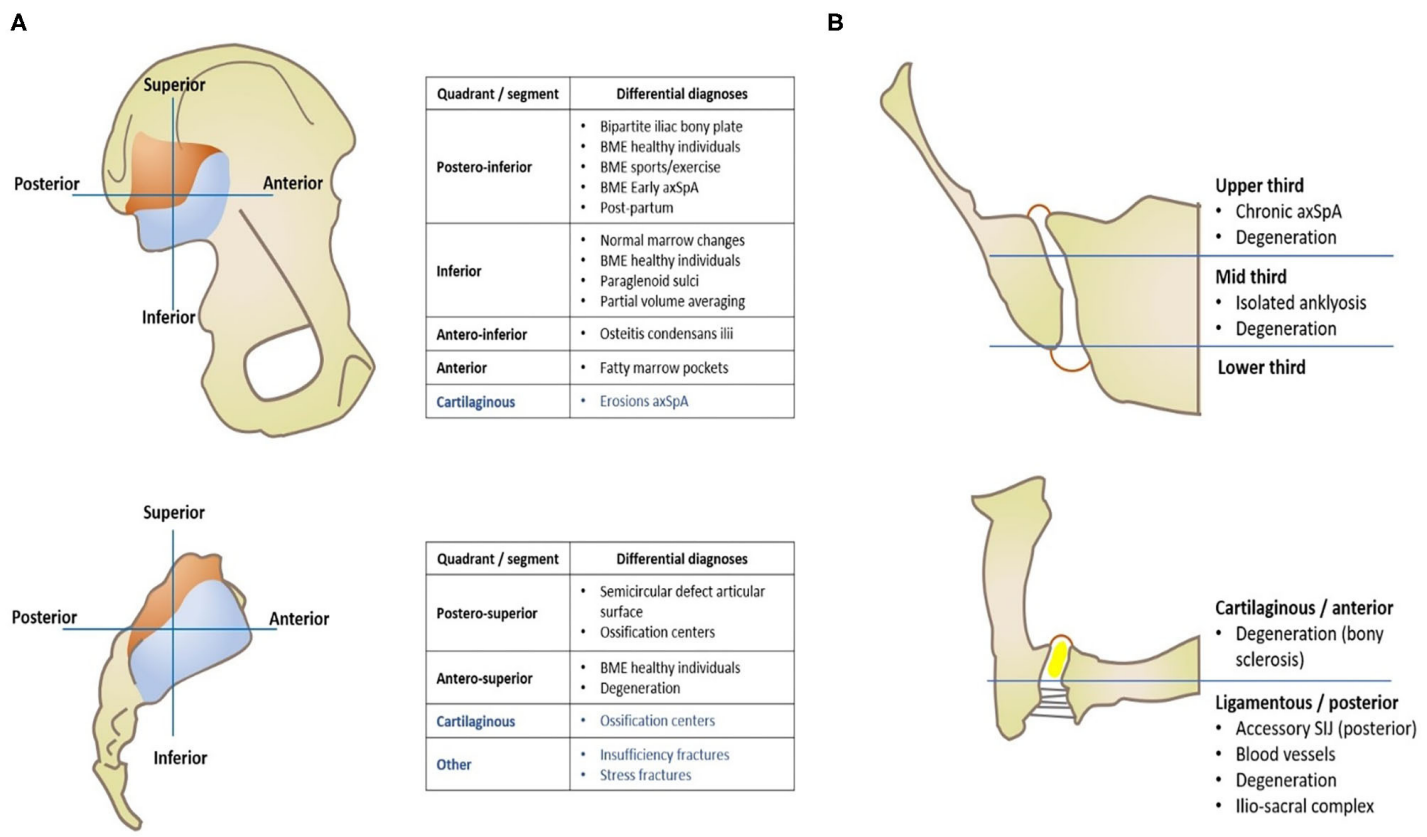
Imaging of the sacroiliac joint—Topographic distribution of main anatomical variants and pathological conditions that mimic axSpA, separated by quadrants of each articular surface **(A)** and orthogonal planes **(B)**, namely coronal oblique (right upper image) and axial oblique (right lower image).

MRI evaluation of the SIJ can be quite challenging even for experienced radiologists, due to several pitfalls. Being familiar with the main imaging findings and terminology of axSpA (Table 1) as well as knowing the topographic distribution of common and uncommon conditions involving the SIJ is key to establishing a confident diagnosis (Figures 1A,B).

**Table 1:** Imaging findings of active and chronic changes of the sacroiliac joint and spine in axial spondyloarthritis.

	**Sacroiliac joint**	**Spine**
Active changes	• Bone marrow edema/osteitis • Inflammation at the site of erosion • Synovitis and synovial proliferation • Intra-articular fluid collection • Capsulitis • Enthesitis	• Spondylitis (anterior or posterior corner inflammatory lesions) and enthesitis* • Asseptic spondylodiscitis (Andersson lesion) • Zygoaphophyseal/facet joint arthritis • Costovertebral and costo-transverse joint arthritis • Inflammation of other vertebral elements (e.g., pedicles and spinous processes) • Inflammation of spinal ligaments
Chronic changes	• Cortical bone erosions and pseudo-widening of joint space • Joint space narrowing • Subchondral sclerosis • Fat depositions/collections (including fat deposition in an erosion cavity, also known as “backfill”) • Ankylosis/bone bridging • Juxta-articular osteoporosis	• Syndesmophytes • Ankylosis/bone bridging • Ligament calcifications • Erosions • Sclerotic changes • Fat deposition on vertebral corners and other previously inflamed bone marrow • Osteopenia

* *The terms “Romanus spondylitis” and “shiny corners” have been used in the context of MRI assessment but should be avoided as they were initially described in plain radiographs: “Romanus spondylitis” appears as irregularity and erosion involving the anterior and posterior corners/edges of the vertebral endplates, while “shiny corners” represent reactive sclerosis secondary to inflammatory process*.

In this article, we will review common and uncommon pitfalls, congenital disorders, normal variants and pathological conditions that may mimic spondyloarthritis affecting the axial skeleton.

## Anatomy of the Sacroiliac Joints and the Spine

The SIJ is the largest joint of the axial skeleton and consists of an amphiarthrosis, exhibits restricted mobility and is separated into a ligamentous (posterior) and synovial (anterior) component. The cartilage covering the synovial segment is thicker on the sacral side and, thus, less prone to lesions (6).

The SIJ is lined by a capsule. Several ligaments contribute to its stability and may be affected in axSpA, namely the anterior and posterior sacroiliac ligaments and interosseus ligament connecting the tuberosities of the sacrum and ilium deeply in the ligamentous portion. The intervertebral disc is also an amphiarthrosis and is comprised of an inner core—the *nucleus pulposus*—and an outer fibrous ring—the *annulus fibrosus*. There is also cartilage lining on the superior and inferior vertebral plates that protects the subchondral bone at this level. The inner core is generally spared in axSpA, but the *annulus fibrosus* attaches to the periphery of the vertebral plates where there is no cartilage protection, and interweaves with the anterior and posterior longitudinal ligaments of the spine, working as an enthesis.

Besides the *annulus fibrosus*, several ligaments stabilizing the spine are prone to inflammation at their insertion point, namely the anterior and posterior longitudinal, supraspinous, interspinous, intertransverse ligaments, and *ligamentum flavum*.

## Normal Variants and Pitfalls

In this section we will describe potential anatomical variants and pitfalls of the SIJ and spine that may mimic axSpA findings (Table 2).

**Table 2:** Congenital disorders and normal variants of the sacroiliac joints and spine that mimic axial spondyloarthritis.

**Condition**	**Type**	**Characteristic features**
Blood vessels	–	Location–ligamentous portion of the SIJ, adjacent, adjacent to anatomical variants, lower ilium (partial volume)
Normal marrow changes	–	Location–lower iliac bone Low SPARCC scores
Healthy individuals	–	Location–anterior upper sacrum, posterior lower ilium
Sports/exercise related	–	Topographic distribution overlaps with axSpA
Port-partum	–	Extent and distribution indistinguishable from axSpA Structural changes are rare
Schmorl nodes	–	Location–lower thoracic and upper lumbar vertebrae, along the *nucleus pulposus* axis
Block vertebra	Congenital	Location–cervical segments Other associated conditions
	Acquired	Other findings–post-surgical, degenerative disc disease, advanced axSpA
SIJ normal variants	Iliosacral complex	Location–Ilium opposite posterolateral sacrum, extra-articular, ligamentous portion Other–women
	Paraglenoid sulci	Location–inferior ilium Other–women
	Ossification centers sacral wings	Location–postero-superior border, cartilaginous portion Other–triangular shape
	Bipartite iliac bony plate	Location–postero-inferior segment Other–unilateral, women
	Accessory iliac joints	Location–between iliac and sacral surfaces at posterior joint
	Semicircular defect articular surface	Location–ligamentous portion, postero-superior, focal sacral depression Other–women, bilateral
	Isolated ankylosis	Location–mid-third of the SIJ
Transitional vertebrae/Bertolotti syndrome	–	Variable presentation (Castellvi classification) Types II and IV correlate with symptoms and disc herniation
Spina bifida occulta	–	Location−5th lumbar segment Other–correlation with spondylolysis
Intra-osseous pneumatocyst	–	Location–iliac bone adjacent to SIJ, lumbar or cervical spine
Tarlov cysts	–	Location–sacrum Other–bilateral, women, 40 years-old

### Coil Effect

Technical artifact responsible for artificial hyperintensity of structures near the receiver coils. These structures may be mistaken for bone marrow or soft tissue edema. Such findings, however, can be distinguished from true inflammatory changes due to their topographic distribution, which is predominantly peri-articular in the latter scenario.

Inadequate fat suppression is higher in patients with higher body mass index (BMI). Radial k-space sampling, an imaging reconstruction technique utilized in MRI data acquisition that is relatively insensitive to motion artifacts, seems to have a positive impact on image quality in such patients (7). Another solution might be to change saturation techniques, from a spectral pre-saturation of fat signal to a short tau inversion recovery (STIR) sequence, which homogeneously suppresses fat, with the caveat of reducing overall signal.

The type of coil also seems to have a significant impact on image quality, more so when combined with the correct sequence in reducing artifacts. This combination yields the best inter-observer agreement for bone marrow edema (BME) detection and lowest number of doubtful BME zones (8).

### Phase-Encoded Motion Artifacts

Motion artifact may occur due to vessels, intestinal motion and patient motion. This artifact may cause blurring or a hyperintense image superimposed at or adjacent to the SIJ and mimic BME. Cross-reference between two perpendicular planes may allow avoidance of overcalling lesions (8, 9). Cerebrospinal fluid (CSF) and blood motion artifacts are also common in spine MR imaging (10).

Again, radial k-space sampling offers a higher signal-to-noise ratio and contributes to reduction in motion-related blurring. Application of motion-resistant sequences is also recommended (7). Other techniques may be employed, such as increasing the number of excitations, changing the phase-encoding axis (along the direction of CSF flow) or applying pre-saturation pulses outside the region of interest (11). Repetitive motion from breathing or cardiac motion may be reduced with gating techniques that perform data acquisition at specific intervals.

### Blood Vessels

Blood vessels coursing close to the SIJ and spine along the acquisition plane may simulate bone marrow or soft tissue edema, synovitis or joint fluid on fluid-sensitive sequences. They present as linear hyperintensities along the acquisition plane and may ramify with other vessels on adjacent slices. CSF motion may also be an issue in spine imaging.

Intense vascularization may be seen at the transition between cartilaginous and ligamentous portion of the joint, at the ligamentous portion and adjacent to certain anatomical variants such as the iliosacral complex and the semi-circular sacral defect, which are described in more detail below (12). Vessels can also run along bones.

### Normal Marrow Changes

Red marrow replacement occurs in a centrifugal fashion in individual bones and in a centripetal fashion in the skeleton. The extremities are primarily affected by this physiological aging phenomenon and, by the middle of the third decade (13), most of the bone marrow in long bones has an overall fatty marrow. Individually, conversion into fatty marrow starts in the diaphysis of long bones and progresses to the metaphysis, ultimately converting the distal epiphysis and, lastly, the proximal epiphysis. In the axial skeleton, the pattern of reconversion is less predictable and several patterns have been described in the spine (14). In the pelvis, small pockets of yellow marrow arise in the third decade in the acetabulum and anterior ilium. In the sacrum of male patients there is higher fat content in the lateral masses compared to females (15) and localized aggregates of fat marrow in the lumbar spines and lateral sacral ala are considered normal variants (16).

### General Population and People With Chronic Non-specific Back Pain

Weber et al. showed that 25% of healthy individuals have signs suggestive of sacroiliitis on MRI (17). Similarly, Arnbak et al. found that, in 1,020 unselected individuals, 21% had sacroiliitis on MRI according to ASAS criteria.

Other authors (18) suggested that one fourth of asymptomatic individuals and more than half of women with post-partum back pain without axSpA had MRI positive sacroiliitis according to ASAS criteria. This study also showed that frequent runners have similar findings compared to asymptomatic individuals and that scoring high on a specific scoring system used for axSpA activity (Spondyloarthritis Research Consortium of Canada Scoring System for Sacroiliitis, SPARCC) is rare in healthy individuals and runners. Furthermore, deep lesions are specific for axSpA-related sacroiliitis and BME lesions in healthy individuals are preferentially located in the lower iliac bone.

**Figure 2:**
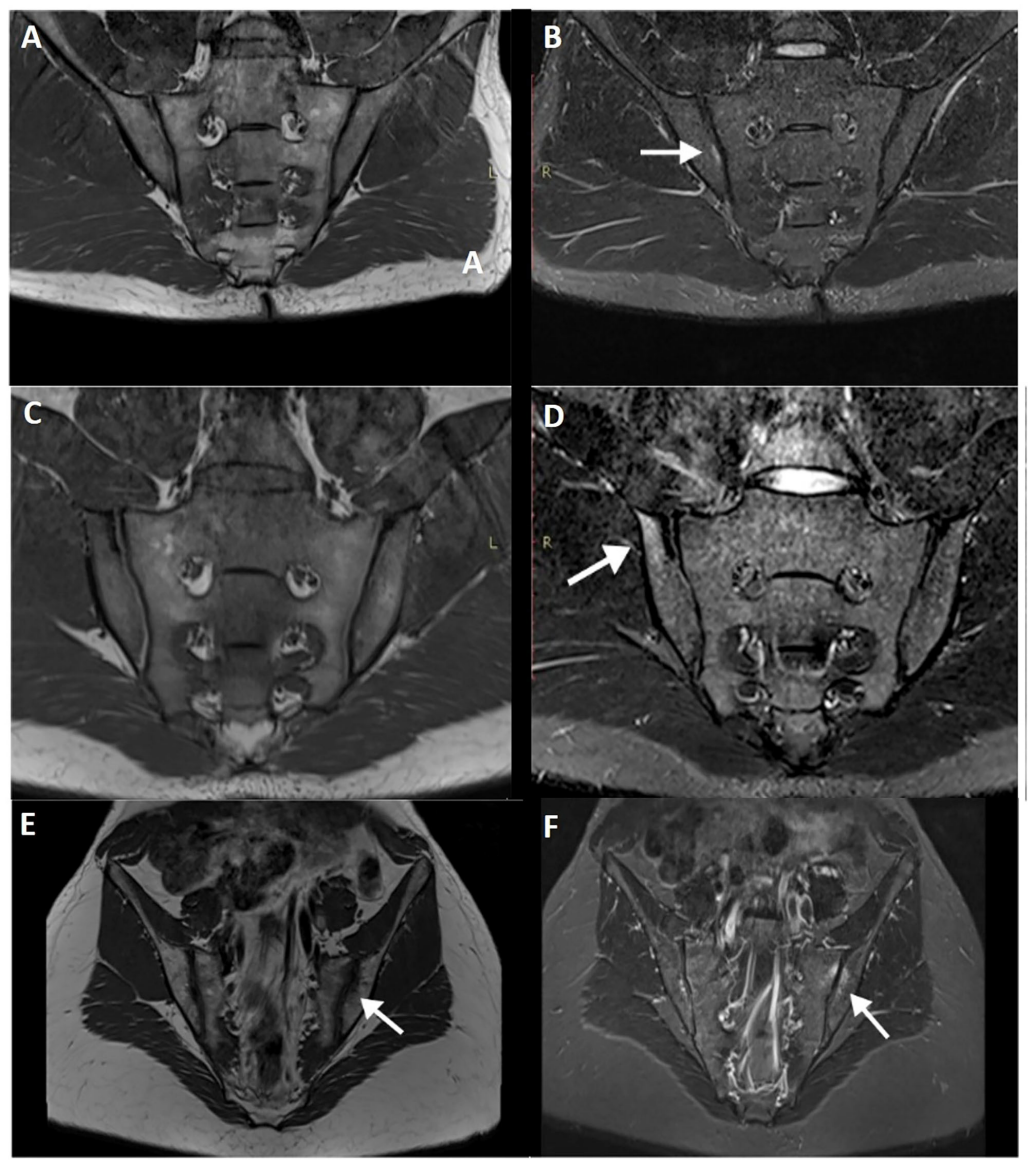
T1WI **(A)** and STIR image **(B)** of a military subject showing a small, peri-articular, area of bone edema (arrow) on the iliac side of the right sacroiliac joint. T1WI **(C,E)** and STIR **(D,F)** images of a post-partum female with bilateral foci of bone edema (arrows) adjacent to the sacroiliac joint.

Indeed, others studies have documented the presence of BME in healthy individuals without any symptoms of low back pain, which does not change in the setting of mechanical stresses or physical exercise (19) (Figures 2A,B). Recently, however, a large population study by Baraliakos et al. (20) confirmed a high prevalence of inflammatory and fatty changes in the SIJ and spine, which increases in frequency with age, suggesting a mechanical factor to their development.

### Sports/Exercise Related BME

Evaluation of MRI lesions in athletes poses a significant challenge when attempting to discriminate healthy individuals from early axSpA. In fact, 30–35% of recreational runners and 41% of elite hockey skaters have shown ASAS criteria for axSpA when evaluated for sacroiliitis (17). Partial volume effect of vascular structures, mechanically triggered BME due to axial strain and normal anatomical variants are thought to be the main reasons for such findings. Applying a complementary semi-axial plane for evaluation seems to significantly reduce ASAS positivity to 20 and 18%, respectively for recreational runners and elite hockey skaters (8).

The two most common portions of the joint affected by BME are the anterior upper sacrum and the posterior lower ilium, the latter associated with partial volume effect of vessels and deep iliac ligament insertion. Unfortunately, it is well-recognized that the early incipient findings of inflammatory changes in axSpA patients show a topographic overlap with BME associated with constitutional features on the dorso-caudal portion of the SIJ, at the posterior lower ilium.

Low-grade BME lesions may indeed have several potential triggers such as mechanical overload or stress, anatomical variations, heavy load work, overweight and post-partum. Discriminative factors that may indicate possible or probable axSpA have not been determined—BME extension alone has not proven to be a relevant criterion (9), but evaluation of extent and topographical pattern might be able to reduce false-positive assessments of ASAS MRI positive sacroiliitis. Assessment of other structural features and active lesions may improve specificity (21, 22).

### Post-partum

Low back pain is common during pregnancy and shortly after birth, typically resolving 6 weeks post-partum. Some patients, however, experience long-standing low back pain more than 6 months after childbirth (23).

Causes for post-partum symptoms are multifactorial and involve mechanical stress and hormonal changes, child and birth characteristics (24). Post-partum SIJ infection is an important differential diagnosis as it accounts for 15% of septic sacroiliitis events; auto-inflammatory conditions may also manifest during pregnancy or after childbirth.

Agten et al. (25) compared the SIJ of post-partum women and women with known axSpA and found no distinguishable features based on extent and distribution, making it difficult to avoid overcalling axSpA in such patients (Figures 2C–F). Presence of structural changes, however, was more frequent in the axSpA group and only rarely found in the post-partum group. Furthermore, pain referral and pain intensity were not correlated with BME in the post-partum group. Importantly, puerperal diastasis of the pubic symphysis and SIJ is physiological to some degree and only in rare situations is associated with complications (26).

Nonetheless, Winter et al. showed positive findings on MRI of post-partum women with back pain, which was consistent with previous data that reported 60% of such patients having SIJ BME lesions on MRI (18, 27).

### Schmorl Nodes

Schmorl nodes correspond to herniation of nucleus material through the endplate of the vertebral bodies into the subchondral bone (28).

Schmorl nodes are usually marginated by a well-defined sclerotic border which may be irregular and are more prevalent in the lower thoracic and upper lumbar segments. The etiology of Schmorl nodes is multifactorial, involving trauma and congenital causes. There is also an association with smoking habits, vertebral body length, and age (28). Patients with Schmorl nodes may be asymptomatic or present with low back pain, and an association with degenerative spine disease and disc degeneration has been established (29). If Schmorl nodes become symptomatic, MRI may demonstrate inflammation and edema in the bone marrow surrounding the Schmorl node. Vertebroplasty has been tried out and proven to be effective and safe when symptoms do not resolve with medical or physical therapy (30, 31).

Acute Schmorl nodes may mimic other inflammatory conditions affecting the spine. Imaging features are of a concentric ring-type edema and involvement of the adjacent end-plate to the herniated node, without diffuse signal abnormalities (32).

### Block Vertebrae

Block vertebrae may be congenital or acquired. Congenital blocked vertebra is generally found in the cervical spine and associated with Klippel-Feil syndrome (short neck, low hair line, and neck movement restriction). Other abnormalities associated with congenital block vertebra include syringomyelia, diastematomyelia, or tethered cord (33).

Acquired vertebral fusion may be a desired surgical outcome in cases of advanced degenerative disc disease or cases of joint instability (34, 35). Also, late-onset ankylosing spondylitis with extensive calcification may lead to bamboo spine due to dystrophic and ligament calcifications so extensive that they merge both endplates of the disc joint. Interbody fusion requires disc removal through a posterior or anterior approach, insertion of a bone graft and/or fusion hardware. The purpose is to achieve an arthrodesis along the disc space. Complications include pseudarthrosis, when bone bridging does not develop or is insufficient. Studies to evaluate post-operative fusion include CT, MRI and bone scintigraphy.

### SIJ Anatomical Variants

Synovial recesses, bony and cartilage clefts that may mimic bone erosion, intense vascularization on the ligamentous portion that enhances avidly and fat infiltration of the sacral bone marrow without pathological significance may be evident on SIJ imaging and are addressed in other sections of this article.

**Figure 3:**
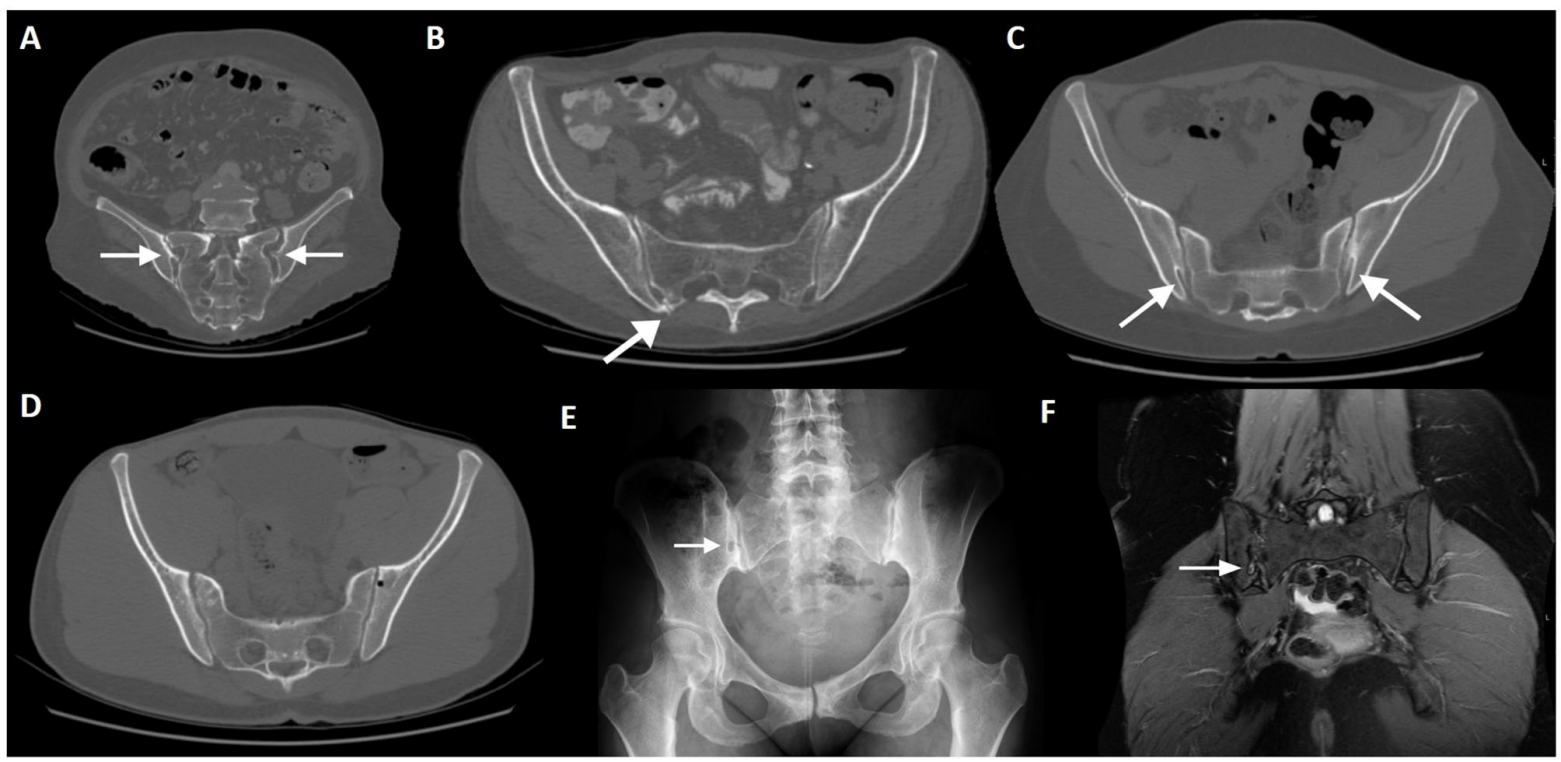
Normal variants and incidental findings of the sacroiliac joint (arrows). CT reconstructions with oblique orientation depict bilateral iliosacral complexes **(A)**, the most common sacroiliac joint variant; right accessory sacroiliac joint **(B)**; bilateral bipartite iliac bony plate **(C)**; left iliac bone pneumatocyst **(D)**. A patient with an incidental finding on the right sacroiliac joint seen on pelvic radiography performed MRI, which revealed an iliac bone cleft filled with fluid **(E,F)**. Note the sclerosis of the symphysis pubis **(E)**, compatible with *osteitis pubis*.

In this section we briefly describe the seven anatomical variants of the SIJ that have been documented to date. The morphology of the sacral and iliac surfaces is well-depicted on CT. The most frequent variants are accessory SIJ and iliosacral complex (Figures 3A,B). These variants are sometimes associated with edematous or structural changes suspected to be mechanical in nature. Positive association between anatomical variations and degenerative changes is somewhat controversial (36, 37). To the best of the authors' knowledge, only one study has analyzed MRI changes in morphological variants of the SIJ (38).

#### Accessory Sacroiliac Joints

The most common variant is an accessory sacroiliac joint (3.6–50%), which is more common in females and has a positive association with increased BMI (38, 39). Accessory SIJ is detected between the iliac and sacral articular surfaces in the posterior aspect of the joint.

It is however not certain if the accessory SIJ are congenital or acquired. In fact, degenerative ankylosis and overall structural changes may masquerade accessory SIJ.

This variation is best depicted on axial slices and is located at the level of the first or second sacral foramen. Signal intensity changes are depicted in a proportion of patients, mostly related to sclerotic or fatty changes, but rarely edematous.

#### Iliosacral Complex

The iliosacral complex corresponds to a marked prominence of the ilium opposite a concave depression of the posterolateral sacrum (40). An iliosacral complex is present in 4% (5.8–11.7%) of individuals and is the second most common anatomical variant and seen bilaterally with slightly increased frequency in women (38). The iliosacral complex is mostly found at the level of the S1 foramen and corresponds to a marked prominence of the ilium projecting to a concavity of the lateral sacrum, in an extra-articular portion of the SIJ (39). This variant is best depicted on coronal images and mainly located between the first and second sacral foramen. Half of cases show prominent vascular structures adjacent to the complex, which may mimic enthesitis.

The interpretation of the SIJ and, specifically, the joint space width, should take into account these variations and the presence of significant extra-articular portions of the ilium and sacrum at different levels. The sacroiliac ligaments insert in such depressions and cavities at the posterior-inferior ilium.

SIJ degeneration is more prevalent in patients with iliosacral complex compared to other morphological variations (36).

#### Other Anatomical Variants

The **ossification centers** of the sacral wings may be persistent in adulthood. They are located at the posterior-superior border of the SIJ, involve the cartilaginous portion of the joint and have a triangular shape.

**Paraglenoid sulci** are small bilateral grooves located in the inferior ilium lateral to the SIJ, more prevalent in women.

Visible at the level of the S2 foramen, **semicircular defects** in the articular surface are represented by an indentation of the ilium toward a mild depression of the sacrum (40). This variant has been described elsewhere as a round defect of the sacrum with or without an opposing iliac defect in the axial plane. It involves the posterior-superior aspect of the ligamentous portion of the joint, is more common in females and mostly bilateral (38).

**Bipartite iliac bony plate** is more frequently unilateral and seen in women, at the posterior-inferior portion of the joint. Crescent-like iliac bony plates have also been described and are seen in 2–5% of patients (36, 39) (Figure 3C).

An **isolated synostosis** has been rarely depicted in two previous studies, in the mid third of the SIJ at the level of the first sacral foramen (38, 41). Absence of structural or inflammatory changes in the remaining SIJ and contralateral side should raise suspicion for an anatomical variation.

### Lumbosacral Transitional Vertebrae and Bertolotti Syndrome

**Figure 4:**
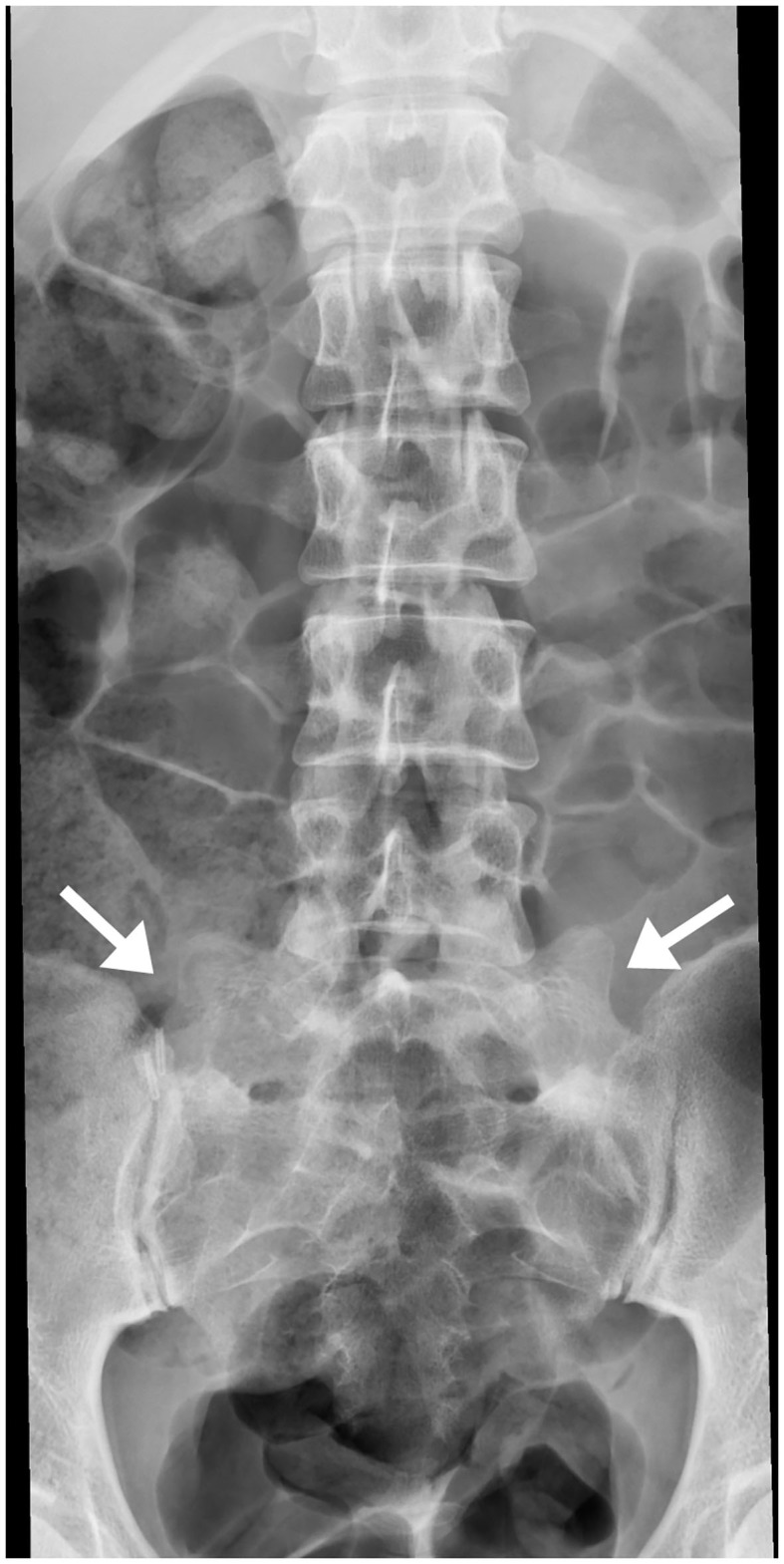
Bilateral transitional vertebra (sacralization of L5), with neo-articulation of both hypertrophic transverse apophyses with the sacrum (arrows).

Lumbosacral transitional vertebra (LSTV) refers to a spectrum of congenital anomalies of the last lumbar and first sacral vertebrae, where an elongated transverse process of the lumbar vertebra articulates or fuses with the first sacral segment (42) (Figure 4). The overall incidence ranges from 4 to 35.6% (43, 44).

Partial articulation and fusion between L5 and S1 lead to limited motion at the lumbosacral joint. This raises mechanical stress to the level above and results in accelerated degeneration of the L4–L5 joint.

The Castellvi classification of LSTV divides in four types (45): (Ia) unilateral, (Ib) dysplastic transverse process with a height >19 mm, (II) incomplete unilateral (a) or bilateral (b) lumbarization/sacralization with engorged transverse process articulating with the sacrum, (III) unilateral (a) or bilateral (b) complete osseous fusion of the engorged transverse processes to the sacrum, (IV) unilateral type II transition with contralateral type III. The most common types in patients with low back pain are IV, IIIb, and II (43). Another study also concluded that LSTV types II and IV positively correlate with prevalence and severity of low back pain (46).

Association between LSVT and low back pain has been termed Bertolotti syndrome, in honor of Dr. Bertolotti who first described the morphological abnormalities, and is an important etiology of low back pain in young patients. LSVT may cause radicular changes and MRI is the examination of choice to evaluate the intervertebral disc as well as the neural foramina (45, 47). An association between LSTV and disc herniation has also been found (48–51).

### Spina bifida occulta

*Spina bifida occulta* (SBO) and LSVT are the most common congenital lumbosacral deformities and involve the 5th lumbar segment (52). SBO is a result of failed fusion of the posterior vertebral elements without affecting the spinal cord or meninges. Its prevalence is estimated between 0.6 and 25% (49).

While SBO occurring in the most frequent segment (L5) does not seem to have any correlation with disc herniation, a previous study has reported an association between SBO of the S1 segment and posterior disc herniation (49). In both pediatric and adult patients, there is a positive correlation of SBO with spondylolysis (53). SBO at other levels is rare (54).

### Intra-Osseous Pneumatocyst and Synovial Cyst

Simple bone pneumatization cysts of the pelvic bones are a common, but poorly understood, innocuous findings on CT (Figure 3D). There have been occasional reports in the literature (55–57) and imaging features include well-circumscribed air-filled round defects of bone with a thin sclerotic rim, usually found adjacent to the SIJ on the iliac bone. They may be an unusual cause of pain that is indistinguishable from other causes of low back pain. In the spine, there are also scarce publications indicating the presence of vertebral pneumatocysts, especially in the lumbar or cervical spine (58).

Synovial cysts, on the other hand, are fluid-filled para-articular lesions that may, but not always, communicate with the joint. These lesions have been described in the SIJ and in the spine, although they are exceedingly rare near the SIJ joint (59, 60) (Figures 3E,F). In the spine, they are most commonly originated from the zygapophyseal joints, in association with degenerative disease.

### Meningeal and Perineural (Tarlov) Cysts

Meningeal cysts may be apparent on pelvic or spinal MRI. These lesions are of unknown origin, and include perineural or Tarlov cysts and arachnoid cysts.

Tarlov cysts, also termed perineural cysts, are common incidental findings on pelvic CT or MRI. They correspond to meningeal dilations of the nerve sheath filled with liquor at the junction of the dorsal ganglion and spinal posterior nerve root at the level of the sacrum. They are typically bilateral, small and asymptomatic and are more common in females at an average age of 40 years (61). Tarlov cysts are visualized on 1–2% of sacral MRIs and 25% are believed to cause symptoms such as low back pain, perineal or lumbar pain, sciatica and rarely, *cauda equina* syndrome (62).

When large, Tarlov cysts may exhibit adjacent bone erosion or endopelvic extension. Enhancement of the cyst should prompt a different diagnosis, such as schwannoma or neurofibroma (63).

Tarlov cysts have originally been described in the sacrum, but they can be found anywhere in the spine (64). Cervical cysts have been increasingly described with MRI. Differential diagnosis includes facet joint cysts and nerve sheath tumors.

Arachnoid cysts arise from the arachnoid membrane through a congenital weakness toward the epidural space. Contrary to Tarlov cysts, the walls or cavities do not contain nerves. They may enlarge and widen the medullary canal or foramina, and cause localized or referred pain (65).

Meningoceles, while not truly cysts, may be confounded with the previous conditions. They constitute protrusions of membrane-lined spinal canal contents through a defect in the column (66). Depending on the herniated content, they may be named myeloceles, myelomeningoceles, or lipomyelomeningoceles. Posterior sacral meningoceles are associated with tethered cord syndrome.

## Pathological Conditions

In this section we will describe several pathological conditions that may mimic axSpA findings (Table 3).

**Table 3:** Pathological conditions that mimic axial spondyloarthritis.

**Condition**	**Type**	**Characteristic features**
Degenerative changes	Spine	Location–weight-bearing axis Other–old age, other degenerative findings, Modic classification
	Sacroiliac joint	Location–ligamentous portion, bone sclerosis of anterior and middle third Other–male, osteophytes, associated with pubic symphysis degeneration
Scheuermann disease	–	Location–thoracolumbar Other–adolescents, Schmorl nodes
*Osteitis condensans illi*	–	Location–iliac side at the ventro-caudal portion Other–bilateral, symmetric, women, middle-age, sclerotic area with triangular configuration, may demonstrate BME below arcuate line, no erosions
DISH and OPLL	DISH	Location–thoracic and lumbar segments, superior non-cartilaginous portion of the SIJ Other–old age, obesity, diabetes mellitus, occasional bridging, appendicular involvement
	OPLL	Location–cervical spine Associated with DISH
Fractures (sacrum/llium/vertebrae)	Acute	Insufficiency–more common at the sacral alae and bilateral, women
	Insufficiency	Stress–clinical history, unilateral and sacral side, no involvement of the subchondral bone, involvement of the pars interarticularis in the spine
	Stress response	Diastasis–clinical history of major pelvic trauma, may have backfill, asymmetry, posterior offset
	Post-trauma inflammatory-like	General–suggestive clinical history, absence of other findings to support axSpA
	SIJ diastasis/incongruence	
Septic arthritis	Familial mediterranean fever/brucellosis	Pronounced edema and other inflammatory osseus and soft tissue changes
	Staphylococcus aureus	
	Pyogenic spondylodiscitis	
	Fungal	
	Tuberculosis	
Metabolic diseases	Idiopathic hypoparathyrodism	–
	Hyperparathyroidism	Other associated findings
	Alkaptonuria	–
	Hypophosphatemic osteomalacia	–
	Paget disease	Bone expansion, cortical thickening, coarsened trabecula
Crystal deposition arthropathy	Gouty sacroiliitis	Location–lumbar spine > rest of the spine or SIJ Other–middle-aged men, perimenopausal women, monoarthritis (mostly lower extremities), SIJ gout is non-specific
	Spinal/Sacro-iliac CPPD	Location–cervical > lumbar segments, atlanto-odontoid joint Other–peripheral arthritis more common, inflammatory flares at the intervertebral endplates
SAPHO syndrome/CRMO	–	Location–clavicles and sternum Other–Extra-musculoskeletal findings, progression from lytic to sclerotic and hypertrophic lesions
Charcot spine	–	Location–thoracolumbar segments Other–spinal cord injury, heterotopic ossification at the elbows and hip, paravertebral masses, bridging osteophytes, degeneration, bone erosions, pseudarthrosis, atrophic to hypertrophic forms
Behçet disease	–	Extra–articular findings, peripheral skeleton most involved, sacroiliitis controversial, atlanto-axial subluxation (anedoctal)
Rheumatoid arthritis	–	SIJ–bilateral and symmetric Other–femoroacetabular joints affected, clinical presentation
Hemoglobinopathies	–	Bone infarctions, bone marrow expansion and hyperplasia, growth disturbance, H-shaped vertebra, red marrow reconversion, extra-musculoskeletal findings
Sarcoidosis	–	Location–throacolumbar segments Other–women, extra-musculoskeletal findings, spinal involvement associated with CNS lesions, lytic, and/or sclerotic lesions in lacework pattern
Early axSpA		Location–dorso-caudal portion of the SIJ (posterior lower ilium)

### Degenerative Changes

#### Sacroiliac Joint

Subchondral BME occurs in early phases of degenerative processes resulting from vascularization of fibrous tissue. It is important to notice the site of edema, since hyperintensity on synovial portions of the SIJ favors inflammatory disease, while ligamentous portion involvement favors degenerative disease.

**Figure 5:**
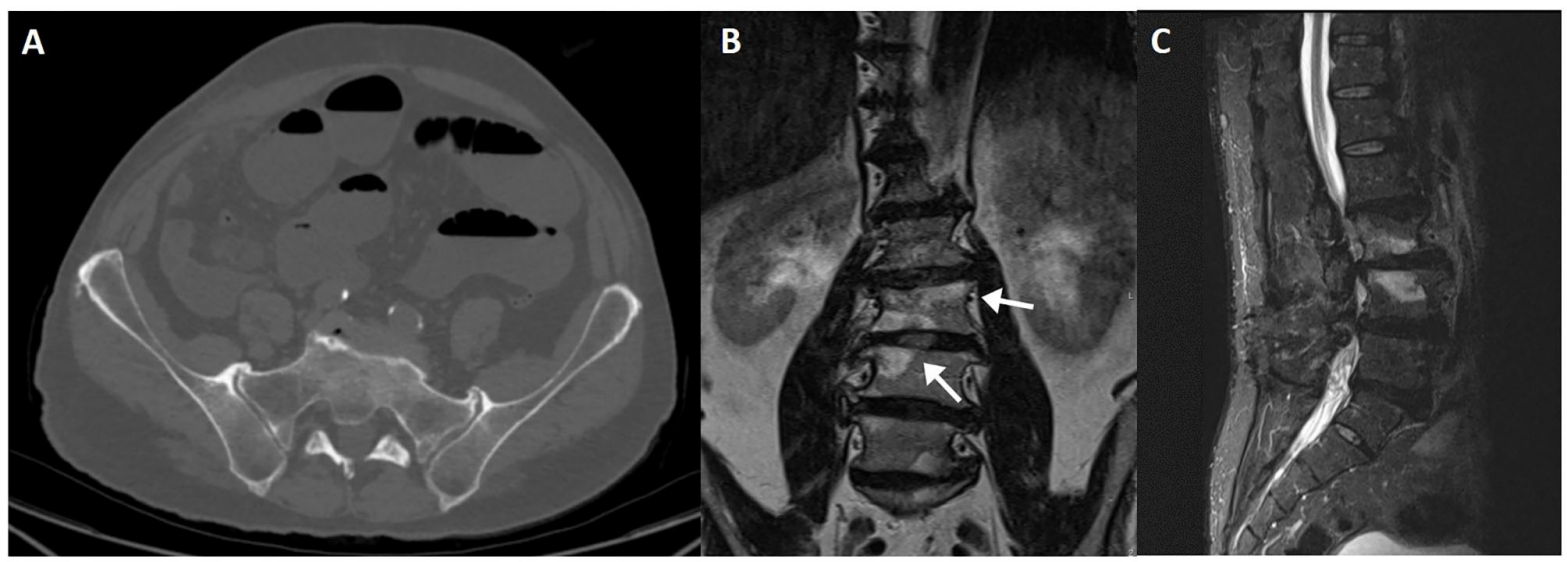
CT axial slice showing degenerative changes of the sacroiliac joint **(A)**, with marginal osteophytes and bone sclerosis. Modic endplate changes at the weight bearing surfaces of the distal lumbar spine (arrows), seen on coronal T1 **(B)** and sagittal fluid-sensitive **(C)** sequences. Bone marrow signal changes are high on T1WI and fat-saturated T2WI, compatible with Modic type 2.

Degenerative changes are more common in men than women and involve osteophyte formation and ankylosis (Figure 5A). A clear connection between CT findings of SIJ degeneration and symptoms has not been found.

SIJ degeneration is common in early decades of life and increases with age. There is a high prevalence of asymptomatic patients with degenerative changes, so caution is recommended when attributing low back pain to SIJ degenerative disease.

SIJ space narrowing is only present in about 25% of patients with degenerative changes. Bone sclerosis is the most common finding, usually at the anterior and middle thirds of the joint and commonly associated with pubic symphysis degeneration.

#### Spine

Degenerative disease of the spine may be confounded with acute or chronic inflammatory changes due to axSpA (67, 68). Progression of intervertebral disc degeneration follows an MRI classification (Modic) that compounds three stages analogous to the Andersson lesions seen in ankylosing spondylitis (AS) (69). Other structures of the spine are usually affected, such as the atlanto-occipital joints, atlanto-odontoid joint, facet joints and the *ligamentum flavum* (70).

The main differences of Modic lesions compared to inflammatory lesions is their topographic location (along the main weight-bearing axis), clinical context (old age and associated with other degenerative findings) and lab work (Figures 5B,C). A multidimensional approach usually suffices to establish the correct diagnosis.

### Scheuermann Disease

Also termed juvenile kyphosis, Scheuermann disease (SD) is the most common cause of symptomatic structural thoraco-lumbar hyperkyphosis in adolescents (13–16 years-old) (71). Its etiology is unknown but several theories have been proposed, such as impaired collagen fibril formation due to changes in growth hormone levels with consequent weakening of the vertebral endplates (72). A strong genetic background has also been reported in recent studies (72). Radiological criteria for establishing SD is not consistent in the literature—some authors describe anterior wedging >5° in at least three adjacent vertebral bodies; others include wedging in one or two vertebral bodies, changes in vertebral endplate, narrowed disc space and anterior Schmorl nodules. An atypical form has been described by Heithoff et al. (73) in the presence of three of the following findings–narrowed disc space, disc dehydration, endplate irregularity, anterior vertebral body edge wedging and Schmorl nodules.

Degenerative disease of the spine is typically present in young patients with SD, namely spondylosis, spondylolisthesis, endplate irregularity and narrowed disc space (with or without associated disc herniation).

### Osteitis condensans ilii

*Osteitis condensans ilii* (OCI) is typically seen in middle-aged women in whom it manifests as sclerotic areas, mainly in the iliac bone, with relatively normal joint spaces, occurring symmetrically and bilaterally at the ventral-caudal portion of the SIJ (74). Its cause is largely unknown but the most accepted hypothesis is that of a mechanical stress, given that such condition is more commonly observed in patients who have given birth, albeit not exclusive.

Radiographs may demonstrate bilateral triangular sclerosis of the iliac wing surface at the SIJ, but osteitis can be unilateral. *Osteitis condensans ilii* is usually asymptomatic, but may present as non-inflammatory chronic back or hip pain.

**Figure 6:**
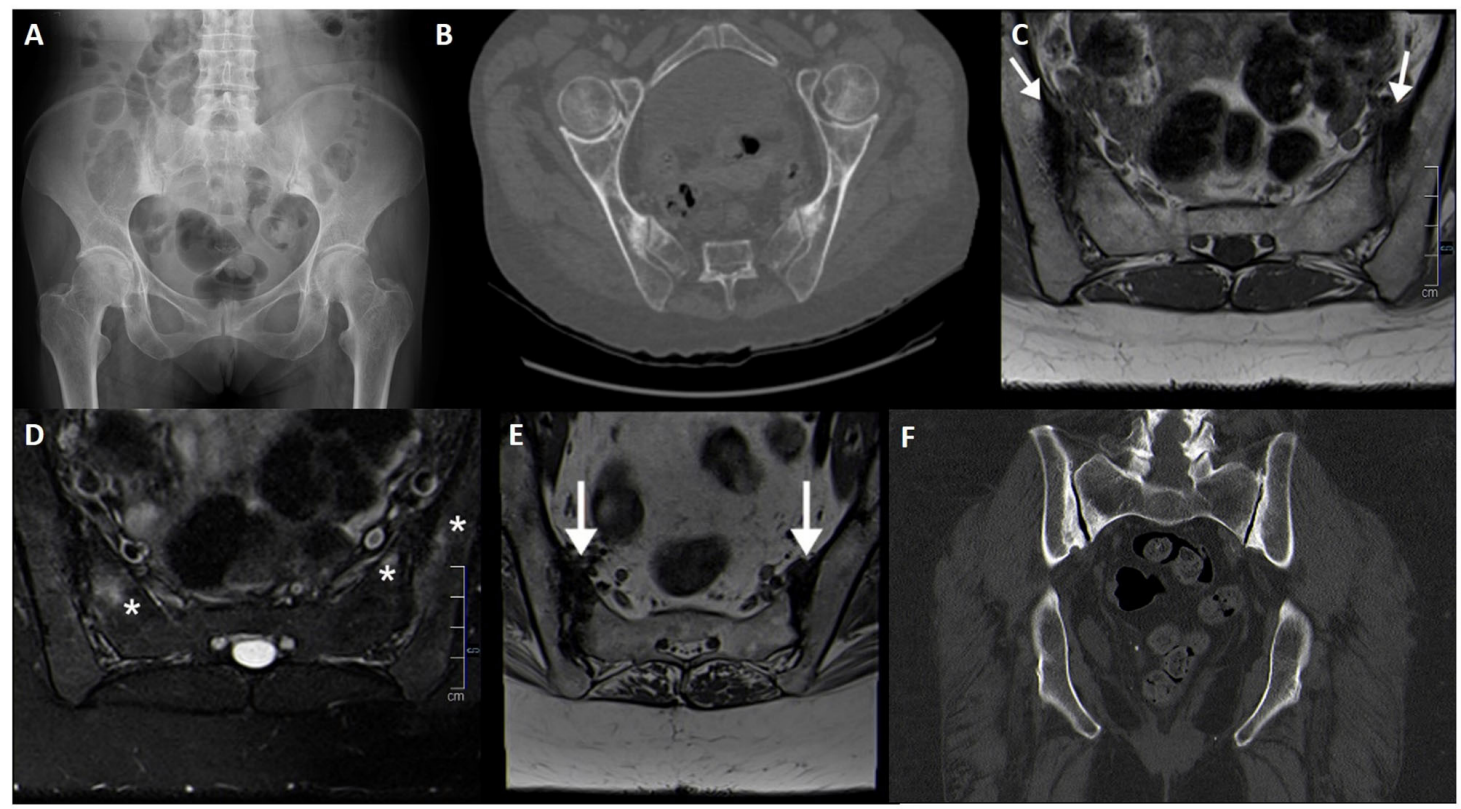
Fifty seven-year-old female patient with bilateral *osteitis condensans ilii* evident on pelvis radiography **(A)** and CT **(B)**. Another patient, with post-partum bilateral bone marrow edema of the sacroiliac joint and sclerotic changes compatible with *osteitis condensans ilii* (arrows, asterisks), shown on MRI sequences **(C–E)** and CT **(F)**.

Differential diagnosis with inflammatory conditions is possible due to lack of erosions, joint space narrowing, ligament calcifications or bone bridging. Sclerosis is present in both groups, but is more pominent in OCI patients. Nonetheless, it has been shown that this condition demonstrates BME on MRI in a significant portion of patients (75), which ranges from mild to as high as vessel signal intensity. BME from OCI is seen in a continuous distribution pattern centered in the ventral-cartilaginous joint part of the ilium and spreads beneath the arcuate line, while BME from axSpA may be scattered and preferentially located at the dorsal-cartilaginous part of the joint and rarely spreads to the marrow beneath the arcuate line (Figures 6A–F).

Some axSpA specific parameters are also present in OCI patients, such as HLA-B27 positivity, inflammatory back pain, and peripheral and extra-articular manifestations, albeit in a smaller proportion of patients (76, 77). Erosions are almost exclusively seen in axSpA patients, especially when multiple.

### Diffuse Idiopathic Skeletal Hyperostosis and Posterior Longitudinal Ligament Calcification

**Figure 7:**
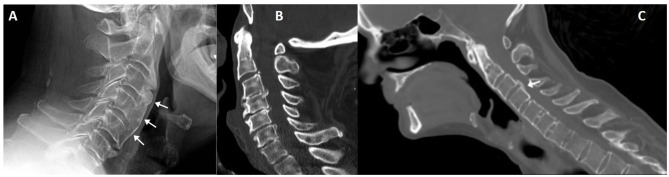
Lateral cervical radiography of a patient with cervical undulating anterior longitudinal ligament ossification (arrows), compatible with DISH **(A)**. Another patient **(B)** with cervical DISH and ossification of the posterior longitudinal ligament; differential diagnosis with posterior syndesmophytes is not always straightforward, as seen in a CT sagittal reconstruction of a patient with ankylosing spondylitis [**(C)**, arrow].

Diffuse idiopathic skeletal hyperostosis (DISH) is characterized by undulating or flowing ossifications along the anterior column of the vertebrae, but also affecting ligaments, tendons, joint capsule, and periosteum, with relative preservation of the disc spaces and absence of radiographic changes associated with degenerative disease (78). It affects older, obese and diabetic patients with increased incidence and may involve any segment of the vertebral column, with affinity to the thoracic and lumbar segments (79) (Figure 7A). Preferential involvement of the superior non-cartilaginous portion of the SIJ is seen, with occasional bridging. There is no sacroiliitis or facet ankylosis. Bone mineral density of the affected segments is also maintained (80, 81).

Radiographs are generally sufficient to make the diagnosis. Other structures beside the axial skeleton might be involved and support the diagnosis, such as the iliac crest, ischial tuberosities, femoral trochanters and the non-articular portion of the patella, with decreasing order of frequency (82). In such places, extensive wavy calcifications are found where ligaments, tendons and capsules attach to bone.

CT and MRI are reserved for whenever there is suspicion of complications, such as dysphagia, nerve compression or fracture (83). Special care should be taken to assess for fractures, which may occur with minor trauma and have a characteristic “carrot stick” appearance that can compress the spinal cord (84, 85).

DISH may be seen in association with posterior longitudinal ligament ossification (OPLL). In fact, both conditions are frequently seen together in nearly half of patients. OPLL predominantly affects the cervical spine and has a more sinister course as it is adjacent to the spinal canal (Figure 7B). It may, however, be difficult to differentiate from a posterior syndesmophyte, as shown in Figure 7C in a patient with ankylosing spondylitis.

### Trauma

#### Insufficiency Fractures and Stress Reaction

**Figure 8:**
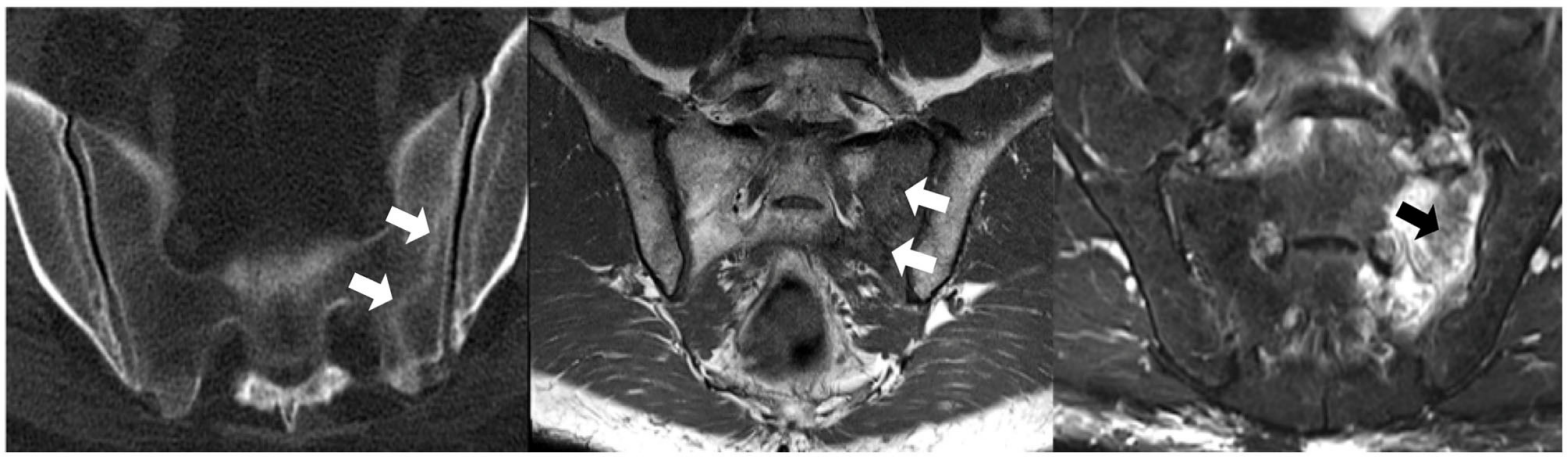
CT (left), T1WI (middle), and fat-saturated PD (right) MRI of a patient with sacral insufficiency fractures (arrows). T1WI shows diffuse slightly hypointense signal of the left sacral wing and a linear hypointensity compatible with a sacral fracture; corresponding fat-saturated PD image documents marked bone marrow edema.

There are mainly two types of sacral fractures—insufficiency and fatigue fractures. Insufficiency fractures are more common and occur with minimal trauma in an osteoporotic bone, are frequently bilateral and have a higher incidence in women (86, 87) (Figure 8).

Sacral stress (or fatigue) fractures are seen in athletes and frequent runners. A stress reaction or fracture may be documented by MRI as a unilateral, sacral side BME without involvement of the subchondral bone. A vertical fracture line within the affected sacrum may be seen and raise suspicion for the diagnosis.

Sacral insufficiency fractures are common (1–1.8%, as high as 5% in some series) and underdiagnosed as a source of low back pain (88). Likewise, vertebral osteoporotic fractures constitute a significant cause of low back pain and disability.

Etiologies include a weakened bone (osteoporosis, steroid-induced osteopenia, infiltrative disease), SIJ pathology (e.g., rheumatoid arthritis) with energy transfer to the sacrum, post-menopause and pelvic radiation. Paget disease, hyperparathyroidism and post-partum sacral fractures have also been reported. Interestingly, 1.6% of regular runners have sacral injuries (89). Mean age of presentation is 70–75 years.

Radiographs are usually unremarkable (20–38% sensitivity, 12.5% with visible fracture line), but when present, fractures are more often seen in the sacral ala (88). Some articles report a sensitivity approaching 0% (90). MRI is the examination of choice given its higher sensitivity, and shows BME.

Fractures involving the spine are more common in the pedicles and *pars interarticularis*, the latter ultimately leading to spondylolysis. Spondylolysis is one of the most common causes of low back pain in young athletes and may be present in up to 47% of symptomatic patients from this group (91).

A radiographic sign of spondylolysis is lateral deviation of the spinous process of the affected level, due to rotation toward the shorter laminae. Radiographs are, nonetheless, limited in documenting this condition and are most useful at depicting spondylolisthesis, which may be another sign of accompanying spondylolysis. CT is the gold standard for detailing bone morphology and detecting pars defects. MRI has a good correlation with CT and SPECT imaging (87). An MRI grading for spondylolysis characterization has been developed (92).

Pedicle stress fractures are also commonly seen among athletes, but may arise as a complication of laminectomy, scoliosis interventions and spine fusion (93, 94). Prevalence in the population is unknown and pathophysiology is controversial. Radiographs may show sclerosis of the pedicles, but other imaging methods are more sensitive, such as MRI or SPECT (91).

#### Post-traumatic Inflammatory-Like Arthritis

Inflammatory-like structural changes of the SIJ have been described in patients after major pelvic trauma, namely fracture or diastasis (95). Clinical symptoms may be of inflammatory or mechanical nature. It is uncertain whether these findings support the theory that axSpA may be triggered through traumatic events or are short-term and self-limited events. Backfill (fat deposition in an erosion cavity), a specific sign of axSpA seen on MRI, has been documented in post-traumatic SIJ diastasis, but may represent a physiological event of bone remodeling in unstable SIJ (95).

SIJ trauma with intra-articular step-off has not been linked to inflammatory-like structural changes.

#### Sacroiliac Joint Laxity and Diastasis

The SIJ space shows important variations depending on the location where it is measured. A joint space under 2 mm is considered pathologically reduced (frequently due to degeneration) (96, 97). However, a detailed anatomy of the SIJ is relevant to avoid erroneous measurements. It is important to take into account that anatomical variants, which are not infrequent, have an impact on SIJ width measurements.

AxSpA affecting the SIJ may produce joint space widening (so-called pseudo-widening) due to cartilage or bone erosions, as well as joint-space narrowing, due to bone remodeling, bridging, and ankylosis. Other conditions described in this article may produce similar findings in the same manner (e.g., erosions in hyperparathyroidism) or in a different fashion (e.g., cartilage wear in degenerative changes). Furthermore, knowledge of patient history is essential; history of trauma to the pelvic ring might cause SIJ diastasis, especially in open-book fractures where the anterior ligaments are torn (98). In these cases, asymmetric widening, evidence of a posterior offset and absence of other findings hint at the probable diagnosis (39, 97).

Sacroiliac joint laxity and hypermobility has been described and may lead to joint instability, disturbance of mechanical loading and development of symptoms.

### Septic Arthritis and Spine Infections

**Figure 9:**
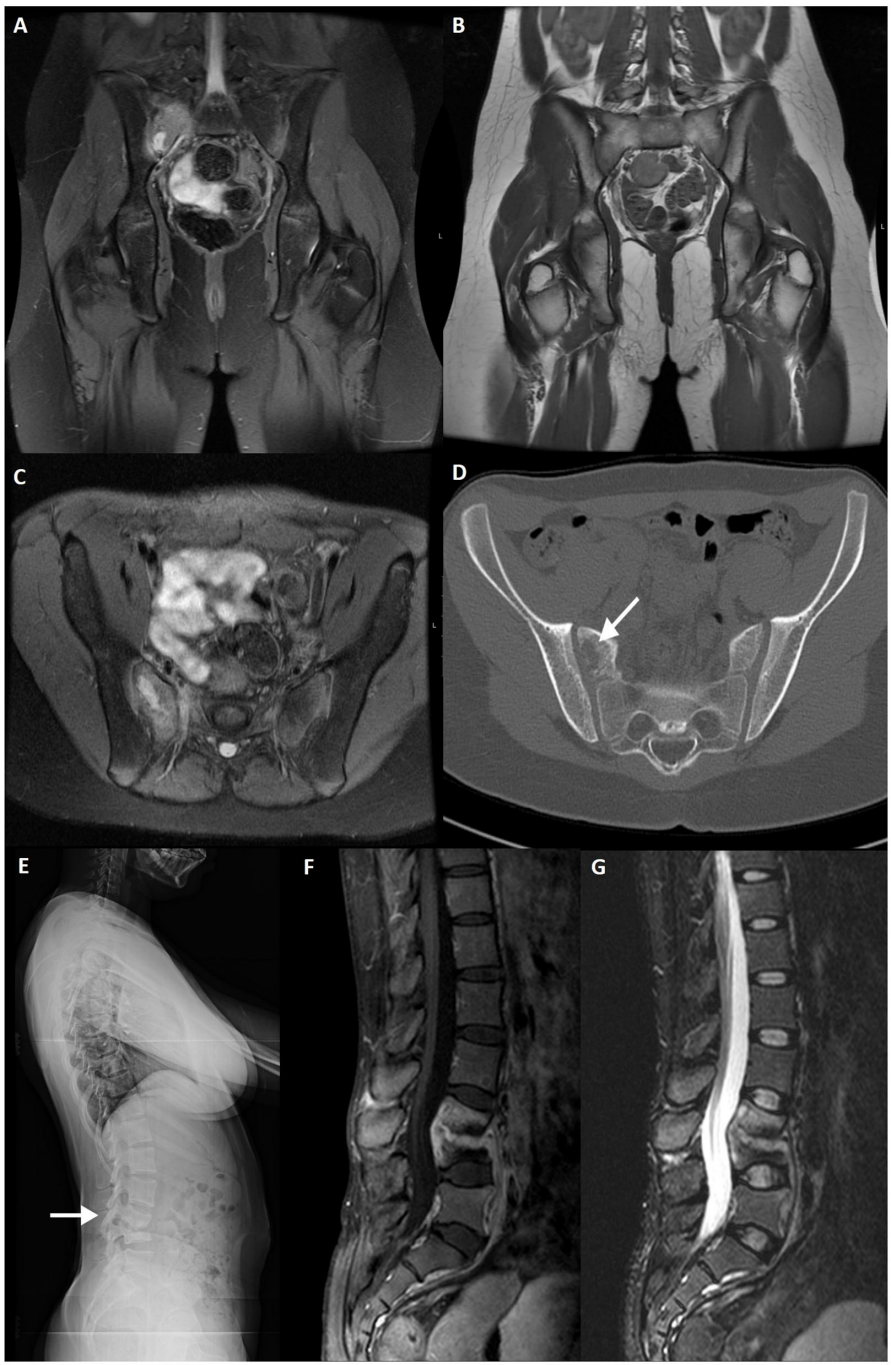
Fat-saturated PD **(A)** and T1WI **(B)** coronal slices, fat-saturated PD **(C)** axial and CT axial slices **(D)** of a 12-year-old female patient with proven *Streptococcus* spp. osteomyelitis of the right sacrum (arrow). A lytic lesion is seen adjacent to the right sacroiliac joint. Lateral lumbar radiography **(E)**, post-contrast fat-saturated T1WI **(F)** and TIRM **(G)** sagittal slices of a 26-year-old female patient with confirmed tuberculous spondylodiscitis of L3–L4 segment (arrow).

SIJ infections arise most often from blood-borne pathogens; erosions of the SIJ may be seen associated with osteomyelitis or soft-tissue abscess (99) (Figures 9A–D). Bacterial forms of SIJ infection may occur through different routes, namely hematogenous, contiguous spread, direct inoculation, or post-surgical. Joint aspiration is often necessary for diagnosis, but clinical and laboratory investigations, aided by CT and MRI showing suggestive findings (see below) may suffice in the presence of a suggestive clinical context. Juxta-articular bone demineralization, considered the earliest finding of infectious sacroiliitis, can be seen on CT. Soft tissue involvement and unilaterality also help in diagnosis (100).

In specific subsets of patients, certain agents might be suspected. Drug addicts are susceptible to infection caused by rare organisms, such as *Klebisella, Enterobacter, Streptococcus, Candida albicans*, and *Pseudomonas spp*.

Facet joint infection is an increasingly recognized entity arising from non-hematogenous sources such as respiratory or genitourinary infections and interventional procedures. Clinical symptoms are similar to spondylodiscitis but generally unilateral erosive bone changes, thickening of the *ligamentum flavum* and obliteration of fat planes may be inconspicuous on CT and only detected on MRI.

Spine infection should be suspected in the clinical setting of new or worsening back pain and fever, intravenous access or hemodialysis, recent bacteremia, endocarditis, intravenous drug abuse or new neurologic deficits (100, 101). It starts as an endplate infection which progresses to discitis. Subtle endplate edema may be the very earliest signs of spondylodiscitis (102). Edema or fluid in the psoas musculature, termed MRI psoas sign, is another finding consistent with early spondylodiscitis (103).

#### Pyogenic Spondylodiscitis

Pyogenic spondylodiscitis is typically centered at the disc space, but may manifest in the bony spinal column and ligaments of the extradural spine. Hematogenous spread is the main route of infection, through arterial supply or paravertebral venous plexus (104). The most common causative agent is *Staphylococcus aureus* (105). Disease has a higher incidence in diabetic and male patients and has an anatomical predilection for the lumbar spine.

In adults, infection spreads from the anterior vertebral body to the remaining body, endplates and adjacent discs. Spread to the paraspinal soft-tissues is common. Documentation of spinal abscesses is particularly relevant as it constitutes an emergency (106). Pediatric patients still have a robust arterial anastomotic network which protects the bone, but the disc is more vulnerable and highly vascularized, thus making it the primary site of infection.

Pyogenic spondylodiscitis reduces disc height and shows hyperintensity on fluid-sensitive sequences that is distinct from the normal hydrated disc pattern. The disc also enhances after gadolinium administration. Bone surface irregularity, destruction and enhancement of the endplates and vertebral bodies is also typical. Extension to the epidural and paravertebral spaces with development of inflammatory swelling, phlegmon, or abscesses is possible (107).

Hyperparathyroidism, neuropathic arthropathy, acute Schmorl nodes, SAPHO syndrome, AS and tumors are non-infectious mimics that may resemble pyogenic spondylodiscitis. Of note, tumor lesions never cross the disc space and the disc height is generally preserved.

#### Familial Mediterranean Fever—Brucellosis

Brucellosis is the most common zoonotic infection worldwide (108). Gram negative bacteria have affinity for the SIJ and up to 35–37% of patients with brucellosis have SIJ involvement, usually unilateral.

The most common manifestations of brucella infection are musculoskeletal and include arthralgia, myalgia and low back pain. Although sacroiliitis is less common than spondylitis, it is still a diagnostic consideration in specific clinical settings.

Brucellosis can mimic axSpA and even fulfill ASAS classification criteria for axial or peripheral SpA (99, 108, 109), when assessing for clinical, laboratory and imaging findings. The most important MRI changes are BME and bone erosions in SIJ. Compared to axSpA patients, BME in brucellosis has higher T2-intensity and usually crosses anatomical borders to affect adjacent muscles. Backfill is also documented, but resolves with antibiotic treatment.

#### Fungal Spondylodiscitis

Fungal spondylodiscitis is a rare occurrence, but incidence has increased over the years due to increase in immunocompromised patients (110). The most common agent is *Candida albicans*, followed by *Aspergillus fumigatus*. Diagnosis is multidisciplinary but the gold standard is histological or culture confirmation from tissue samples. The most affected segments remain the lower thoracic and lumbar spine. Imaging is non-specific and mimics pyogenic or tuberculous infection.

#### Tuberculous Spondylodiscitis

Spinal tuberculosis (TbS) is a common form of extrapulmonary tuberculosis and accounts for 50% of musculoskeletal tuberculosis cases (111) (Figures 9E–G). Clinical presentation is non-specific long-standing back pain, which may be investigated only after onset of neurological deficits and bone deformities. Nevertheless, in countries with a high prevalence of tuberculosis clinicians should be alerted to this possibility and include it at an early stage in the differential diagnoses, thus avoiding misdiagnosis.

Radiographs show loss of endplate margin definition, kyphotic changes, narrowing of the intervertebral disc space and calcified paravertebral masses.

TbS may resemble other pyogenic infections involving the disc. Some findings that favor TbS include: larger collections, cold abscesses adjacent to the affected spine, thoracolumbar junction, no/less involvement of the disc space, skip lesions involving multiple ligaments through subligamentous spread and whole vertebral body or posterior involvement (107, 112). Suggestion of a degenerative nature relates to the presence of vacuum phenomenon, preservation of the cortical boundaries, lack of soft-tissue involvement and stability of radiological findings.

Modic type 1 degeneration may mimic TbS, but contrast enhancement of active degenerative lesions is milder compared to TbS.

### SAPHO Syndrome and CRMO

**Figure 10:**
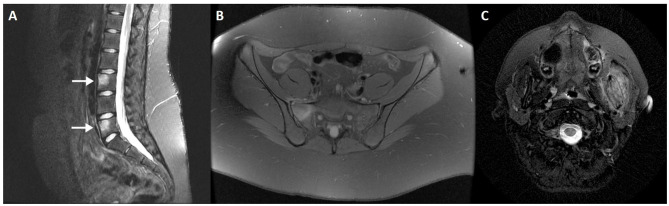
Fat-saturated T2WI sagittal MRI sequence **(A)** of the lumbar spine in a 16-year-old female patient with CRMO (arrows), mimicking corner inflammatory lesions. Fat-saturated PD axial slice **(B)** of the same patient depicting involvement of the right SIJ. Fat-saturated T2WI axial slice **(C)** of the neck shows involvement of the left mandibular ramus.

Synovitis, acne, pustulosis, hyperostosis and osteitis (SAPHO) syndrome is a rare auto-inflammatory condition that shares musculoskeletal and cutaneous manifestations (113). Chronic recurrent multifocal osteomyelitis (CRMO) is considered the pediatric counterpart of SAPHO syndrome, arising from sterile osteomyelitis (Figures 10A–C). In CRMO, cutaneous involvement is less common and long bones are more affected compared to the sternum and clavicles in SAPHO syndrome.

SAPHO syndrome has been considered an umbrella term including several idiopathic disorders sharing similar clinical and radiological features, namely CRMO in children and adolescents.

Radiographs are generally normal in early-stage CRMO but may eventually show small lytic lesions which become progressively more sclerotic (114). This condition may be self-limited and eventually resolve or lead to marked hyperostosis (115).

Whole-body MRI is the gold standard modality for evaluation of SAPHO and CRMO, due to its sensitivity and lack of radiation (116). The most frequent findings are:

- Lytic lesions in early-stage
- Sclerosis, bony expansion or mixed lytic and sclerotic changes in later-stage
- Pathological or compression fractures, with associated deformities in fluid-sensitive sequences
- Bone expansion / hyperostosis in late-stage

Spinal SAPHO syndrome may mimic infectious spondylodiscitis (117). However, absence of soft-tissue masses and epidural involvement as well as the presence of anterior vertebral corner erosions differentiate it from an infectious nature. Nonetheless, bone biopsy is necessary to exclude infection or malignancy.

### Metabolic Diseases

Certain metabolic diseases may show imaging changes suggestive of axSpA.

Hypoparathyroidism occasionally courses with syndesmophytosis and para-spinal ligament calcifications that resembles psoriatic arthropathy (118, 119). Other radiographic findings include diffuse increased bone mass, osteosclerosis of the calvarium with narrowed diploic space (120).

**Figure 11:**
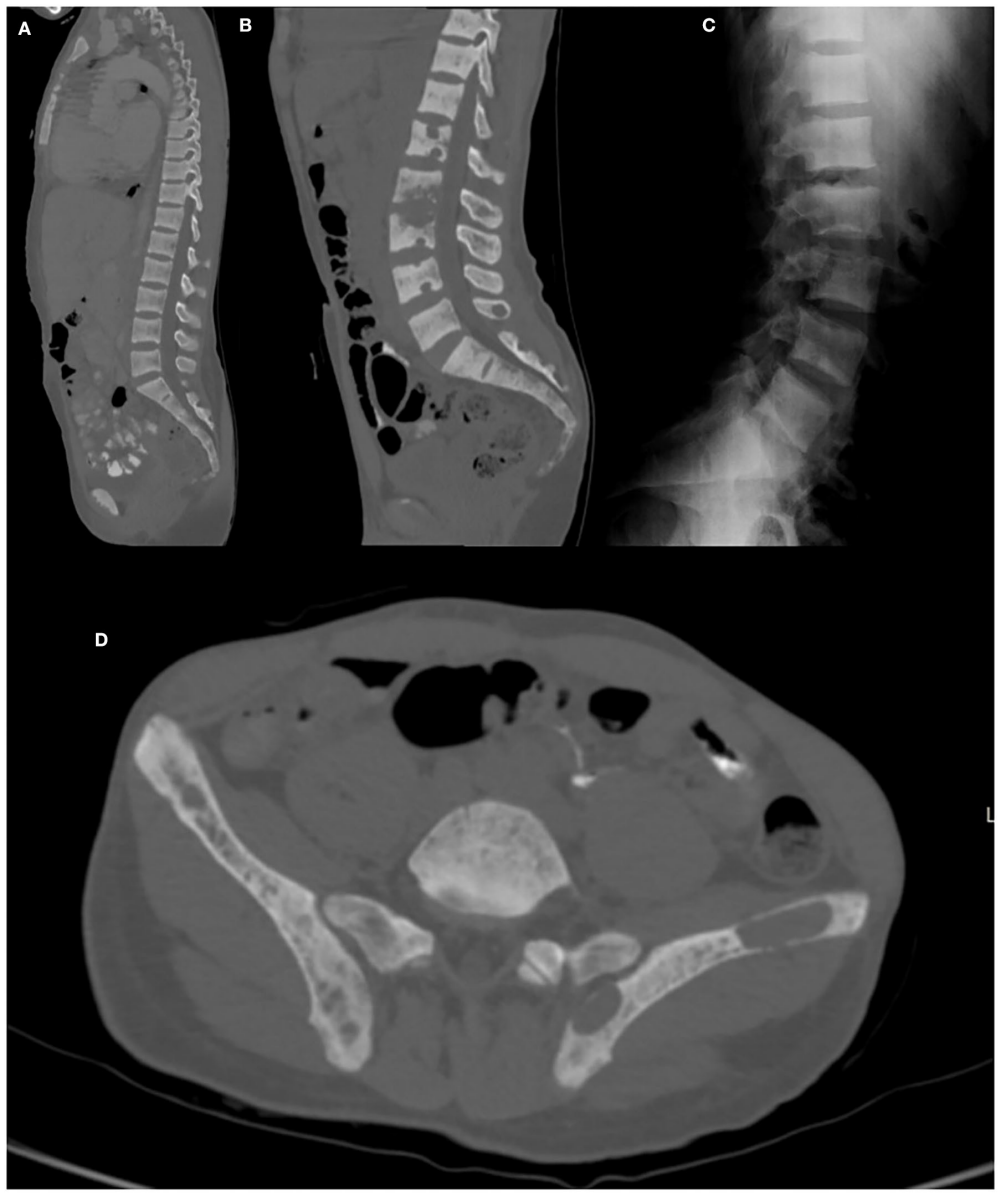
CT sagittal reconstruction of the dorsal and lumbar spine **(A,B)** of a patient with renal osteodystrophy depicts abnormal bone turnover and mineralization, with diffuse osteosclerosis and multiple areas of subperiosteal resorption. Lateral lumbar spine radiography **(C)** shows the characteristic “rugger jersey” spine, with alternating bands of increased and normal bone density of the vertebral bodies. Note a large brown tumor of the left iliac bone on CT **(D)**.

Hyperparathyroidism may course with subchondral bone resorption anywhere in the axial skeleton (Figures 11A–D). Musculoskeletal changes in hyperparathyroidism are most common in the hands (95%) (121), with pathognomonic subperiosteal bone resorption on the radial side of the middle phalanges of the middle and index fingers (122). Acro-osteolysis may also be seen, due to bone resorption of the distal phalanges. Other forms of bone resorption have been described, such as subligamentous, intracortical, subchondral, endosteal, or subtendinous locations. Subperiosteal resorption may also affect the ribs, tooth sockets, humerus, femur, and tibia. Subchondral resorption in particular can occur in any joint, along the interphalangeal and metacarpophalangeal joints, acromioclavicular joint, SIJ, and sternoclavicular joint. Subtendinous resorption is more typically found in the calcaneus, clavicle, proximal humerus and femur, ischial tuberosity, and anterior-inferior iliac spine. BME and other active and chronic features may be seen in the SIJ, but with the same frequency as that seen in healthy individuals and lower than in patients with axSpA (123).

Changes in hyperparathyroidism resemble those from AS but are distinguished due to abscence of joint space narrowing and less pronounced articular surface irregularities.

Hypophosphatasia is a rare genetic disorder that results in accumulation of pyrophosphate, an inhibitor of bone mineralization, and development of hypophosphatemic osteomalacia. Radiological findings are similar to rickets and osteomalacia and vary according to age of presentation (121).

#### Paget Disease

**Figure 12:**
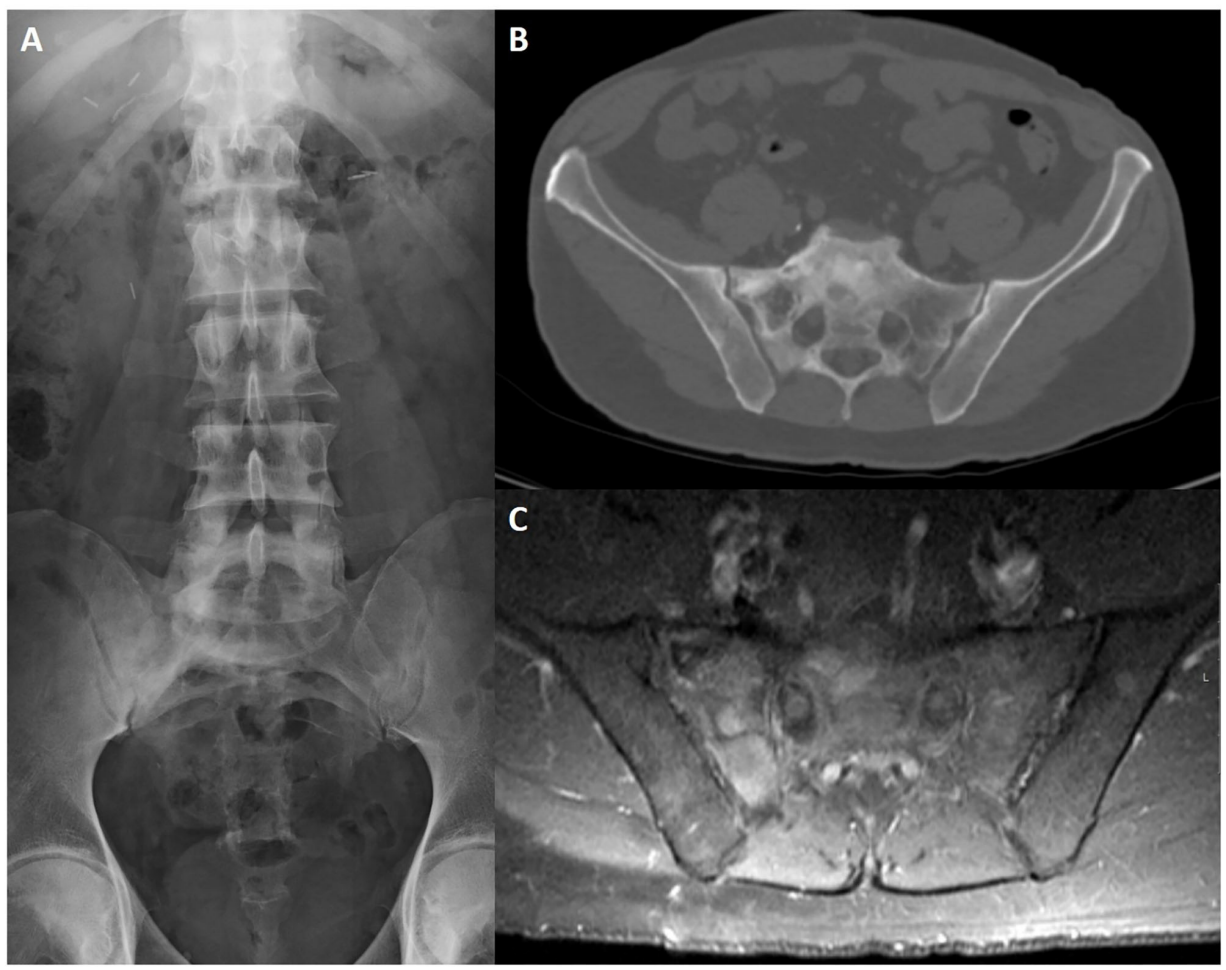
Lumbosacral radiography **(A)**, CT axial slice **(B)** and post-contrast fat-saturated T1WI **(C)** of a 65-year-old male patient with Paget disease of the sacrum and right iliac bone. Typical findings include an expanded bone with coarsened trabecular pattern and sclerotic changes that are more evident on conventional radiography. There is increased uptake after intravenous contrast injection **(C)**.

Paget disease (PD) of bone, also known as *osteitis deformans* and described for the first time in 1877 by Sir James Paget, is a chronic skeletal disorder characterized by abnormal and excessive bone turnover (124) (Figures 12A–C).

PD is more prevalent among Anglo-Saxon descendants, males and patients over 50 years old. Prevalence increases with age but incidence has been declining over the last 20 years.

PD is a disease of largely unknown causes, but the role of environmental factors on a background of genetic susceptibility have been increasingly recognized and are proposed by some authors as the most likely etiology. Viruses seem to be the main causative agent, since patients present with intranuclear and intracytoplasmic inclusion bodies in osteoclasts and giant osteoclasts (classic features of virus infection).

The disease course can be divided in three main phases (lytic, mixed, and sclerotic), although some authors describe a fourth inactive phase. All phases can occur simultaneously in the same patient at different sites (125).

Most patients will be asymptomatic at the time of diagnosis, explaining why the disease is most often discovered incidentally. Symptoms, when present, vary depending on the distribution of the disease, with pain being the major complaint. Fractures are the most common complication (126).

Distribution is generally asymmetric, most commonly affecting the lower extremities with a slight tendency for the right-side. The most common affected sites are the lumbar spine (L4 and L5), pelvis, sacrum, femur, and calvarium (127).

PD typically begins with bone destruction translated into a lytic phase, which is characterized by intense osteoclastic activity displayed as osteolysis. Progression of the disease into a mixed lytic and sclerotic phase usually occurs with time. The four cardinal features of this stage include:

- Advancing edge of osteolysis
- Coarsening and thickening of bone trabeculae along the stress lines
- Cortical thickening
- Osseous widening/bone expansion (pathognomonic)

In long bones, early-stage PD will appear as an advancing edge of osteolysis which begins in the subchondral bone and extends to the metaphysis and diaphysis, giving the characteristic “flame” or “blade of grass” configuration.

In the spine, cortical thickening along the four margins of the vertebral body cortexes is usually seen, giving a “picture-frame appearance.”

Osteosclerotic phase is characterized by increased bone density. Coarsening of the trabeculae and cortical thickening, associated with marked widening and enlargement of bones, will be apparent in long bones and pelvis. Diffuse sclerosis of the vertebral body is typical in this stage, giving the appearance of ivory vertebra. Involvement of the spine may affect one vertebral level, multiple levels, or even all vertebral segments. Posterior vertebral elements may also be affected.

PD can also invade the intervertebral disc and articular surfaces directly, extend to ligaments, and cause ligamentous ossification.

### Crystal Deposition Arthropathies

#### Gouty Sacroiliitis

Gout is a common metabolic disease that frequently affects middle-aged men and postmenopausal women. The most frequent manifestation is monoarthritis secondary to tophi deposition, more common at the lower extremities but eventually involving any appendicular or axial joint (128). Initial involvement of the first metatarsal-phalangeal joint may be followed by tarsal, ankle, knee, finger, wrist and elbow involvement and, less frequently, shoulders, hips, spine, and SIJ. Both the spine and SIJ may be affected, but the most common location is the lumbar spine.

Sacroiliac gout has an incidence of 7–17% (129) and symptoms are non-specific, mimicking other inflammatory, or infectious conditions. In fact, this condition is frequently misdiagnosed as AS. A correct diagnosis may require biopsy or aspiration with polarized microscopy evaluation to reveal the monosodium urate crystals.

Imaging findings are non-specific and CT is the preferred method of choice for detection of subcutaneous tophi and structural changes suggesting gouty arthritis. Dual energy CT may directly visualize and quantify crystal deposition (130).

#### Spinal/Sacro-iliac CPPD

Calcium pyrophosphate dihydrate crystal deposition (CPPD) may be secondary to metabolic disorders such as hemochromatosis, hyperparathyroidism and hypomagnesemia, or less commonly, a monogenic familial disease. CPPD may occur in cartilage and fibrocartilaginous joints, a process termed chondrocalcinosis. Other structures may be affected by CPPD, such as ligaments and tendons, the *nucleus pulposus* and *annulus fibrosus* of the intervertebral disc. CPPD is predominantly a peripheral arthritis, but spinal involvement has been documented (131).

A destructive arthropathy affecting the cervical and, less commonly, lumbar segments is seen and, among these segments, the transverse ligament of the atlas and, thus, the atlanto-odontoid joint is the most frequent (132, 133). CPPD deposits in the peri-odontoid region may lead to a condition called crown dens syndrome when associated with acute symptoms (134, 135). Furthermore, severe retro-odontoid deposits may generate cervical myelopathy due to spinal cord compression.

Aseptic discitis is a well-known complication of CPPD arthropathy in the axial skeleton and causes recurrent inflammatory flares (136) (vertebral endplate erosions, intervertebral disc narrowing, and gadolinium enhancement of the disc and endplate lesions.) A percutaneous biopsy of the affected structures may be necessary to exclude infection or other etiologies.

The SIJ is rarely affected but may also be responsible for acute flares. Degenerative changes in asymptomatic individuals and, occasionally, destructive changes have been described. Again, such changes are non-specific and other diagnoses should be excluded.

MRI has poor sensitivity to detect CPPD deposits, but reveals inflammatory changes of the endplates and SIJ.

### Bone Tumors

**Figure 13:**
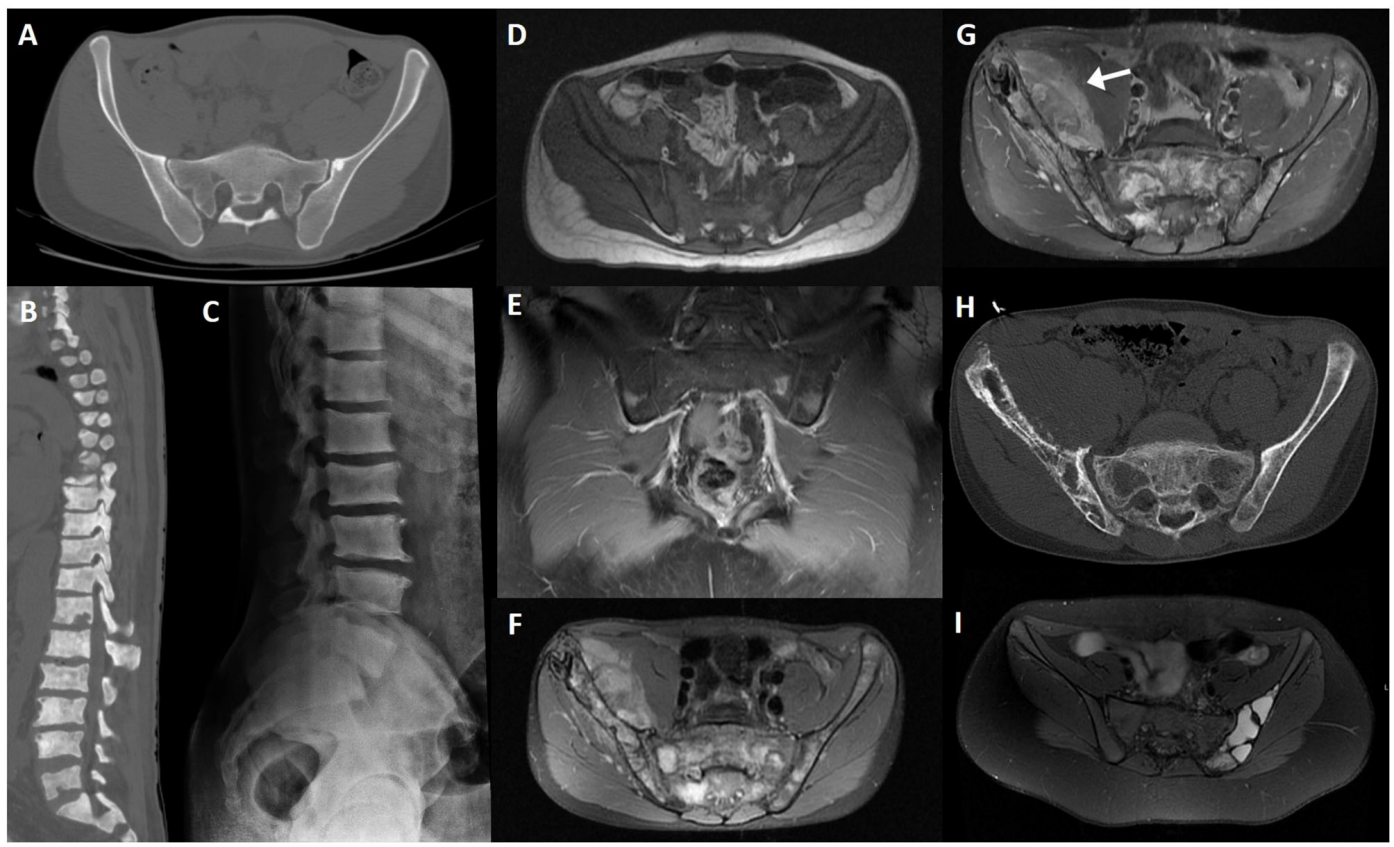
CT axial slice **(A)** of an iliac bone enostosis mimicking peri-articular sclerosis; CT sagittal reconstruction **(B)** of the dorsal and lumbar spine in a patient with diffuse osteoblastic metastasis due to prostate cancer; lateral lumbar radiograph of the same patient **(C)**; T1WI axial slice **(D)**; and post-contrast fat-saturated T1WI coronal slice **(E)** of a patient with leukemic infiltration of the sacrum and iliac bones, showing diffuse bone marrow T1 hypointensity due to tumoral infiltration and multifocal patchy uptake, respectively; fat-saturated PD **(F)**, post-contrast fat-saturated T1WI **(G)** MRI and CT axial slice **(H)** of a 18-year-old male patient with Ewing sarcoma; fat-saturated PD **(I)** MRI sequence of an aneurysmatic bone cyst of the left iliac bone.

Diagnosis of primary or secondary bone tumors is usually straightforward, but they may appear like BME on MRI, especially when infiltrative in nature (Lodwick type IC, II and III) (137). Their typical location, however, is not near the SIJ and lesions are better demarcated after endovenous contrast injection. The sacrum is a common site for multiple myeloma, plasmacytoma and metastasis involvement. Vertebrae are also a frequent site of metastases (Figures 13A–I).

#### Benign Primary Tumors–Pelvis

Most benign tumors of the pelvis occur before the age of 40. In general, benign tumors have geographical well-defined borders (Lodwick type IA or IB) and may be expansile, unlike inflammatory conditions, but occasionally appear more aggressive and have blurred borders (Lodwick type IC) (137).

In an initial assessment, benign tumors involving the posterior sacrum may be confounded with other entities, especially when seen in a young patient.

Osteochondromas are among the most common benign tumors and have a characteristic appearance of a cartilage-covered bony projection, usually pointing away from the nearby joint (138).

Giant cell tumors (GCT) are more common in women than men and usually appear in the third and fourth decades of life, after physeal closure. They can have locally aggressive features and high vascularity. A small subset of GCT is malignant (5–10%) (138). The sacrum is the most common site of involvement in the axial skeleton and GCT is the second most common tumor involving this bone, following chordoma. Imaging findings include a lytic soft-tissue mass with increased vascularity, occasionally crossing the SIJ, with low signal intensity on T1 and heterogeneous on T2 (hypointensity of the solid component) weighted-imaging. There is no periostitis or bone matrix formation; GCT may have an associated aneurysmal bone cyst, with evidence of fluid-fluid levels.

Aneurysmal bone cysts are typically lytic and well-circumscribed, expansile with thinning of the cortex. Variable T1 and T2 weighted imaging signal intensity due to the presence of blood products with different ages is common; fluid-fluid levels are characteristic, but not specific.

#### Benign Primary Tumors–Spine

Vertebral hemangiomas are common spinal tumors and typically multiple (139). Hemangiomas may have distinct presentations, but the most common appearance on MRI is T1 and T2 weighted imaging hyperintensity owing to their hamartomatous nature with vascular and fatty components.

Other tumors involving the spine that are more frequently seen include eosinophilic granuloma, osteoblastoma, GCT, aneurysmatic bone cyst, and osteochondroma. A detailed description goes beyond the scope of this article and is expertly addressed elsewhere (139).

#### Bone Marrow Infiltrative Lesions—Lymphoma, Leukemia, Multiple Myeloma, Plasmacytoma, Ewing Sarcoma, Metastasis

A wide range of conditions affect marrow composition either through infiltration or component replacement. Neoplastic and myeloproliferative processes increase cellularity and have distinct imaging patterns.

In general, tumor cells have long T1 values (decreased signal) and variable T2 values. Imaging has a role not only in diagnosis, but also evaluation of remission or progression of disease. Infiltrative marrow has a decreased T1 signal intensity, with the exception of melanoma and some cases of myeloma (140). T2 signal is more variable.

Lymphoma, leukemia, plasma cell myeloma, primary bone neoplasms, and metastatic disease may have either a focal or diffuse distribution in the bone marrow. The spine and pelvis are among the most common bones involved in these conditions (141, 142).

Spinal metastases generally appear on the posterior-superior aspect of the vertebras, in the vertebral body, and destruction of a pedicle is not an uncommon finding. Focal lytic metastases demonstrate decreased signal on T1 compared to muscle or disc, and increased signal on T2 compared to normal marrow. Blastic lesions have decreased signal on T1 and T2 weighted imaging (143). Post-contrast T1 weighted imaging sequences demonstrate mild to moderate enhancement.

MRI may document other signs of an infiltrative process, namely vertebral collapse, intra-spinal soft-tissue and cord compression, muscle infiltration or lymph node enlargement.

Both Hodgkin and non-Hodgkin lymphoma tend to affect the spine (144) in a focal nodular pattern. Signal intensity on conventional MRI sequences is similar to metastatic disease from solid neoplasms, with abnormal lymphomatous marrow enhancement. Vertebral collapse and soft-tissue mass may be found.

Ewing sarcoma affecting the axial skeleton is most common in the ribs and pelvis (138).

#### Multiple Myeloma and Solitary Plasmacytoma

Multiple myeloma (MM) is a plasma cell dyscrasia with proliferation and accumulation of monoclonal plasma cells (145).

Conventional radiography has a low sensitivity for detection of lytic lesions, and new advances in the last 2 decades have increased the role of MRI and PET CT to evaluate bone marrow infiltration in early and late stages.

The most frequently used conventional sequences are T1 and T2 weighted acquisitions with and without fat suppression for qualitative determination of bone marrow composition and mineralized matrix (146). Dynamic contrast-enhanced and diffusion-weighted imaging also play a role in diagnosis.

Lesions appear hypointense on T1 and relatively hyperintense on fat-suppressed T2 due to high cellularity and water amount. MM favorably affects the axial skeleton (lower thoracic and lumbar spine) and pelvis, but also the ribs, shoulders, skull, and proximal femurs. Patterns of infiltration may differ—no change, focal infiltration, diffuse disease, salt-and-pepper involvement or combined. Almost one third of patients exhibit normal appearing marrow signal on T1 and fat-suppressed T2 weighted imaging. MM lesions have high contrast-enhancement due to neo-angiogenesis, with washout. High signal on high *b*-value images correspond to bone marrow infiltration.

Red bone marrow, usually more pronounced in young individuals, tends to have the same signal intensity changes compared to MM infiltrated bone marrow. Contrast-enhancement curves may vary, and Dixon techniques may be applied to distinguish red bone marrow hyperplasia from an infiltrating lesion (147).

Mean age of patients with MM is over 50 years. Subchondral geodes, schwannomas, Schmorl nodules and scar tissue from bone marrow biopsy may simulate MM on conventional MRI.

Plasmacytoma lesions generally have hypointense signal on T1 and hyperintense signal on T2 weighted imaging. Post-contrast sequences demonstrate intense enhancement. These lesions are expansile and may show a “mini-brain” appearance on axial images. Distinction from other entities such as metastasis, lymphoma or leukemia may be challenging (148).

#### Other Malignant Primary Tumors

Chordoma is the most common primary sacral tumor (149). It is a low-grade malignant tumor arising from notochordal remnants. Imaging shows a heterogenous sacral mass causing bone destruction and expansion. Chondrosarcoma, Ewing sarcoma and osteosarcoma also favor the pelvis, but diagnosis is usually straightforward and a detailed description goes beyond the scope of this article.

### Charcot Arthropathy

Charcot neuroarthropathy of the spine, also called Charcot spine, is progressive destruction of the spinal joint due to innervation abnormalities (98, 150). Charcot spine and heterotopic ossification are possible outcomes of spinal cord injury. Heterotopic ossification occurs most often around the hip or elbow joints (151). Insensitivity to pain with failure to activate muscle contraction is the proposed etiology to these conditions.

The spinal column is involved in 6–21% of patients with neuroarthropathy (152), more often in the lower thoracic (below T10) and lumbar segments (L4–L5) (153). Imaging findings include spinal instability, bridging osteophytes, paravertebral masses, cartilaginous destruction, intervertebral disc degeneration, bone erosion, early face destruction, and pseudarthrosis. CT plays an important role in depiction of most abnormalities, with MRI providing better resolution of the adjacent soft-tissue. Description of an atrophic form and progression to a hypertrophic form may explain differences in presentation.

Spinal fusion is recommended, with high rates of recurrence.

### Sclerosing Dysplasias

Sclerosing bone dysplasias (SBD) are a group of skeletal abnormalities characterized by a wide variety of clinical and radiological presentations. Hereditary SBD include osteopetrosis, pyknodysostosis, osteopathia striata, osteopoikilosis, and progressive diaphyseal dysplasia. There are some non-hereditary forms, namely melorheostosis, intramedullary osteosclerosis and overlap syndromes.

Such conditions manifest with increased bone density that may be diffuse (e.g., osteopetrosis) or focal (e.g., melorheostosis), affecting the periosteum, endosteal cortical lining, or the medullary canal, with variable distribution.

Recognition of SBD may be difficult and such conditions may mimic bone metastasis, metabolic and hematological disorders as well as inflammatory conditions.

A more detailed depiction of the most common conditions goes beyond the scope of this text and is best described elsewhere (154, 155).

### Behçet Disease

Behçet Disease is a multisystem inflammatory disorder mainly manifested by oral and genital aphthous ulcers, skin lesions, and uveitis. Other systems may be less frequently affected, such as the gastrointestinal, central nervous and musculoskeletal systems, as well as the lungs and kidneys. Arthritis and arthralgia are the commonest musculoskeletal findings, interestingly associated with enthesitis in some clusters of patients (156). The chronic and vascular nature of Behçet disease, associated with drug targets that change bone metabolism might lead to reduction in bone mineral density and osteoporosis (157).

Joint manifestations are typically non-erosive, non-deforming and involve the peripheral skeleton in an oligoarticular fashion. The knee is the most frequently affected joint.

In the axial skeleton, prevalence of sacroiliitis in patients with Behçet disease is controversial–some authors report a higher prevalence while others found that there is no significant difference when compared to healthy controls (158, 159). Anecdotal reports have described other forms of axial skeleton involvement, such as atlanto-axial subluxation and instability (160).

### Hemoglobinopathies

Hemoglobinopathies are genetic defects resulting in abnormal structure of the globin chain of hemoglobin molecules, and comprise sickle cell anemia and thalassemia. Sickle cell disease is an autosomal recessive disorder that results in an abnormal morphology of the red blood cell when certain stresses occur. This altered shape leads to vascular stasis, occlusion and infarction.

**Figure 14:**
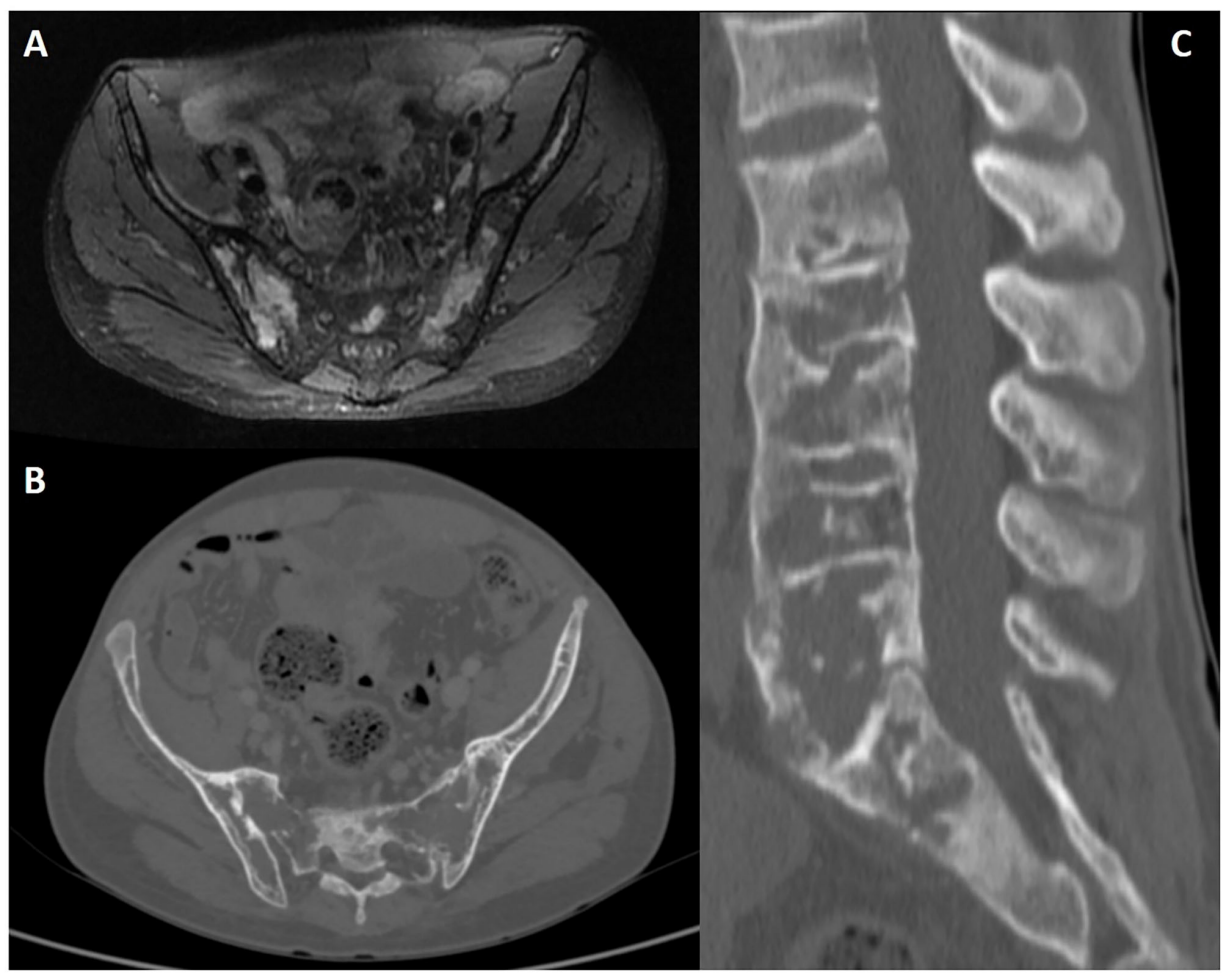
Fat-saturated PD **(A)** MRI sequence, CT axial slice **(B)**, and sagittal lumbar spine reconstruction **(C)** of a 31-year-old male patient with Sickle cell disease and extensive bone marrow changes causing widening of the medullary spaces and thinning of cortical bone.

Musculoskeletal manifestations include bone infarction with or without superimposed infection, bone marrow expansion and hyperplasia (161) (Figures 14A–C). In an acute setting, bone infarcts may have a diffuse appearance, and eventually consolidate into a more sclerotic lesion. On MRI, a serpentine, well-demarcated appearance is seen. Growth disturbances can involve the vertebral bodies and cause decreased height, sclerosis due to bone infarcts and endplate depressions, with the classic H-shaped vertebra (162). Bone marrow hyperplasia on the anterior and posterior borders of the vertebral bodies, accompanied by central depression cause the typical “fish-like” appearance.

Red marrow reconversion in such patients lowers the high T1 signal intensity that is generally seen in fatty marrow of adult patients. Chemical shift imaging and the Dixon technique in particular may play a role in excluding malignant infiltration of affected bone marrow (physiologic red and fatty bone marrow will show a signal drop on out-of-phase images, but malignancy will not).

A detailed description of the musculoskeletal findings in sickle cell anemia and thalassemias goes beyond the scope of this text and has been expertly outlined elsewhere (163).

## Conclusion

Low back pain (LBP) is one of the leading causes of morbidity and poses a significant economic burden in western countries with large numbers of work days lost. The SIJ and lower spine undoubtedly play a fundamental role in the pathogenesis of LBP, even in young and otherwise healthy patients. In fact, the sacrum has been coined the keystone of the pelvis, and deservedly so. Don't let the SIJ fool you—the apparent simplicity of its anatomical and biomechanical properties is only the tip of the iceberg.

AxSpA is an important inflammatory cause of chronic LBP. Clinical evaluation and identification of features suggestive of axial SpA, namely imaging features, is key to early diagnosis and to avoiding misdiagnosis. MRI is of major interest in the assessment of SIJ and the spine when an axSpA diagnosis is suspected. However, clinicians must be aware of imaging mimics and potential pitfalls. For example, although BME is an important imaging finding in axSpA, it is definitely not exclusive of this condition and mimicking changes can also be found in SIJ of healthy subjects, or SIJ presenting with morphological variants, changes related to mechanical stress, degenerative disorders, infection, and neoplastic conditions.

As a general rule of thumb, certain patterns of BME (deep involvement from articular surface, extensive lesions and close relation to other lesion types) as well as the presence of structural lesions, particularly bone erosion, ankylosis, or backfill (or fat deposition in an erosion cavity) increase the likelihood of axSpA. Contextual interpretation of the changes detected on MRI is critical. Ultimately, this information needs to be combined with clinical information, and clinical judgement remains the mainstay for the diagnosis of axSpA.

## Author Contributions

All authors listed have made a substantial, direct and intellectual contribution to the work, and approved it for publication.

## Conflict of Interest

PM has received consulting/speaker's fees from AbbVie, BMS, Celgene, Eli Lilly, Janssen, MSD, Novartis, Pfizer, Roche, and UCB all unrelated to this manuscript. The remaining authors declare that the research was conducted in the absence of any commercial or financial relationships that could be construed as a potential conflict of interest.
